# Integrated Plasma and Bile Metabolomics Based on an UHPLC-Q/TOF-MS and Network Pharmacology Approach to Explore the Potential Mechanism of *Schisandra chinensis*-Protection From Acute Alcoholic Liver Injury

**DOI:** 10.3389/fphar.2019.01543

**Published:** 2020-01-16

**Authors:** Lianlin Su, Jing Mao, Min Hao, Tulin Lu, Chunqin Mao, De Ji, Huangjin Tong, Chenghao Fei

**Affiliations:** ^1^College of Pharmacy, Nanjing University of Chinese Medicine, Nanjing, China; ^2^Nanjing University of Chinese Medicine, The Key Laboratory of Chinese Herbal Medicine Processing of Jiangsu Province, Nanjing, China; ^3^School of Medicine and Life Sciences, Nanjing University of Chinese Medicine, Nanjing, China; ^4^Affiliated Hospital of Integrated Traditional Chinese and Western Medicine, Nanjing University of Chinese Medicine, Nanjing, China; ^5^Affiliated Hospital of Integrated Traditional Chinese and Western Medicine, Jiangsu Province Academy of Traditional Chinese Medicine, Nanjing, China

**Keywords:** *Schisandra chinensis*, alcoholic liver injury, metabolic profiles, UHPLC-Q/TOF-MS, network pharmacology

## Abstract

*Schisandra chinensis* (SC) is a well-known important traditional Chinese medicine (TCM) that has been used to treat liver disease in China for a long time. However, its overall effects and mechanism of action are unclear. The present study aimed to explore the potential mechanism of SC in protection against alcoholic liver injury (ALI). In this research, to enable a full assessment of metabolic changes in ALI in Sprague-Dawley rats and to increase our understanding of physiological changes in normal and pathological states, ultra-high performance liquid chromatography combined with quadrupole time of flight mass spectrometry (UHPLC-Q/TOF-MS) was used to probe potential biomarkers to learn more about ALI and to evaluate the overall effect of SC for ALI in rats. Principal component analysis (PCA) and orthogonal partial least squares discriminant analysis (OPLS-DA) were used to investigate global metabolomic alterations and to evaluate the therapeutic effects of SC in rats. The component–target–pathway network of SC was then constructed on the basis of the network pharmacology, and the liver injury-relevant signaling pathways were thus dissected and validated. The results showed that SC has conspicuous therapeutic efficacy for ALI, as suggested by the results of the pathological section and biochemical index assays, such as those for Alanine aminotransferase (ALT), Aspartate transaminase (AST), Alkaline phosphatase (AKP), γ-glutamyl transferase (γ-GT/GGT), Reactive oxygen species (ROS), and Malondialdehyde (MDA). Furthermore, 21 kinds of potential biomarkers were identified in plasma samples of ALI rats, and 20 kinds of potential biomarkers were identified in their bile samples. The biomarkers were mainly related to inflammation and dysfunctions of amino acids and energy metabolism. The recovery of these dysfunctions partly led to the curative effect of SC on ALI.

## Introduction

*Schisandra chinensis* (Turcz.) Baill (Bei Wuweizi) is a broadly used TCM (Chinese Pharmacopoeia Commission [CPC], 2015a). Modern scientific research has proved that SC contains lignans, essential oils, organic acids, and the chemical composition of vitamins, triterpenoids, sesquiterpene, and polysaccharides etc., and that the lignans are the main active ingredient among them (Cheng et al., 2014; Hu et al., 2014; Zhu et al., 2015). They have beneficial pharmacological action, including a hepatoprotective effect, anti-oxidant, anti-tumor, anti-HIV, and antiviral activity, and antagonistic activity toward platelet-activating factor (Chun et al., 2014; Liu et al., 2014; Xue et al., 2015: Zhong et al., 2015). The separation of 11 lignans from SC, including Deoxyschizandrin, Schisandrin, Schizandrin C, Schisantherin, Schisantherin B, Schizandrin, Schizandrol B, Schisanhenol, Gomisin G, Gomisin J, Angeloylogomisin H., has been achieved in a previous study (Hu et al., 2013).

The attention of researchers all over the world has been attracted by liver diseases, especially cancer. Though surgical removal in cancer is an ideal option, it works in only 10-30% patients (Lau and Lai, 2008). Thus, chemotherapy can be a good alternative choice of treatment. However, its extensive application is confined by the serious damage caused by chemotherapy to many normal organs. A targeted drug delivery system is a good way to reduce side effects and improve therapeutic efficiency. So far, a variety of liver-targeting techniques have been developed, such as microspheres, nanoparticles, cellular carriers, drug-monoclonal antibody conjugates, liposomes, and drug-polymer conjugates (Moses et al., 2003; Tommasi et al., 2007; Li and Wallace, 2008; Ressom et al., 2012). Despite great achievements, the results are not as ideal as expected (Rivera et al., 2002; Sams-Dodd, 2005). Thus, Traditional Chinese medicine (TCM) has attracted more and more attention due to its minor side effects and widespread use in clinical practice.

Metabolomics, which was developed by Nicholson, Lindon, and Holmes (Nicholson et al., 1999; Wang et al., 2004; Huang et al., 2010; Zhou et al., 2012; Liang et al., 2016), could provide a global view of endogenous fluctuations in response to exogenous stimuli, making it a good way to detect a physiopathological response to a drug-, toxin- or disease-induced disturbance. Thus, it has been successfully used for evaluating drug toxicity, making disease diagnoses, and providing biomarkers that are specific for early stages of liver and kidney injury (Beger et al., 2010; Chadeau-Hyam et al., 2010; Chen et al., 2011). The use of a combination of UHPLC-Q and TOF-MS is prevalent because of its high resolution, sensitivity, simplicity, and high throughput, and this approach has been successfully used to investigate the metabolic changes induced by disease and drug toxicity.

Network pharmacology is a new pattern of drug research that analyzes the relationship between drugs, targets, metabolic pathways, and diseases (Hopkins, 2007; Hopkins, 2008). Based on existing data in a database, the establishment of a component–target–pathway–disease network for drugs, and the analysis of the intervention and impact of drugs on the entire disease network, the effects of pharmacodynamic components on certain key targets and their pathways can be predicted and validated. The composition of TCM is diverse, and it has complex network relationships with diseases and targets. Therefore, network pharmacology is quite suitable for the study of the potential mechanism of action of TCM (Su et al., 2013; Ma et al., 2018).

*Schisandra chinensis* is a frequently used material medicine in Chinese patent medicine and TCM prescriptions. Many years of clinical application have shown that the hepatoprotective effect of SC is strong. At present, the mechanism of SC in treating ALI has mostly been studied from the molecular point of view, such as in terms of signal pathways and hepatic drug enzymes (Wang et al., 2014; Chen et al., 2017; Li et al., 2018; Yuan et al., 2018), mainly focusing on the mechanisms of the active components of the SC monomer (Li et al., 2018; Nagappan et al., 2018). There have been few reports on the effect of SC on alcoholic liver injury from the perspective of metabolomics. Although the liver protection given by SC has been known for a long time, the specific target and underlying hepatoprotective mechanism and the effect of SC on hepatoprotective processes are still not clearly demonstrated. Therefore, further investigation is still necessary for clinical application. In this study, the metabolomics method, in combination with the network pharmacology approach, was used to distinguish the variation in the metabolite profile and protective mechanism of SC on ALI.

## Materials and Methods

### Reagents and Materials

#### Drug Materials

**Figure 1:**
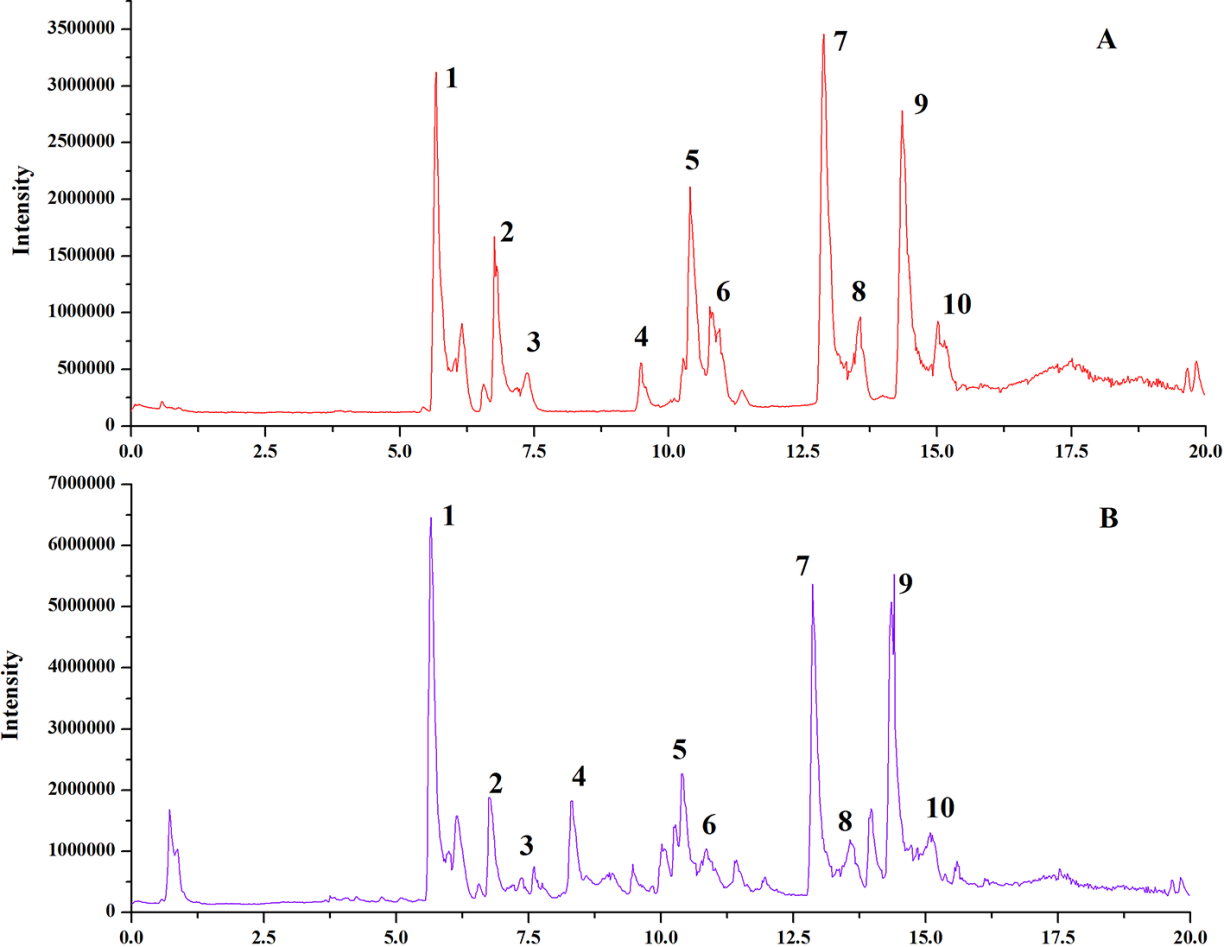
UHPLC-Q/TOF-MS chemical fingerprints (TIC chromatography) of SC. Standard Sample of *Schisandra chinensis* lignans **(A)**, *Schisandra chinensis* ethanol extract **(B)**. Gomisin G (1), Schizandrol (2), Gomisin J (3), Schizandrol B (4), Schisanhenol (5), Schisantherin (6), Schisantherin B (7), Deoxyschizandrin (8), Schizandrin B (9), Schizandrin C (10).

*Schisandra chinensis* (SC) pieces were purchased from Anhui Fengyuan Tongling Herbal Pieces Co. Ltd., Batch number: LOT#140524. The samples were identified as *Schisandra chinensis* (Turcz.) Baill. by Professor Chen Jianwei at Nanjing University of Chinese Medicine. We analyzed the chemical fingerprint (UHPLC-Q/TOF-MS) of SC (**Figure 1**); the specific UHPLC-Q/TOF-MS method used is included in the **Supplementary-1 Information**. Reference standards of Schizandrin, Gomisin J, Schisandrin B, Gomisin G, Schisantherrin A, Deoxyschisandrin, Schisandrin B, and Schisandrin C (purity > 98%) were purchased from the National Institutes for Food and Drug Control (Beijing, China).

#### Main Reagents

In this study, chromatography-grade acetonitrile, formic acid, and leucine-enkephalin were obtained from Merck (St. Louis, MO). Distilled water was disinfected using a Milli-Q Gradient A10 Ultra-pure water meter (Merck Millipore, USA). Red Star Erguotou (56°, a popular kind of Chinese spirit) was purchased from Beijing Red Star Limited by Share Ltd. Sodium carboxymethyl cellulose (CMC-Na) was purchased from Chinese Medicine Group Chemical Reagent Co., Ltd. The ALT, AST, γ-GT/GGT, and MDA assay kits were purchased from the Jiancheng Bioengineering Institute of Nanjing (Nanjing, China). AKP and ROS assay kits were provided by Nanjing Austrian Green Biotechnology Co., Ltd. (Nanjing, China).

### Preparation of SC Solution

A crude slice of the medical material, SC, was extracted twice (1.0 h/time) with 80% ethanol of 8 times volume (W/V). After merging the filtrate and condensing to a certain concentration under reduced pressure, the extractum was dissolved into 0.5% sodium carboxymethyl cellulose (CMC-Na) to obtain the target concentration 2.0 g/mL (the amount of crude herb or pieces). The prepared drugs were stored at 4°C until administration to rats.

### Animal Models and Alcohol-Induced Liver Injury Study

A total of 40 male Sprague-Dawley rats (200 ± 20 g, aged approximately 8 weeks) were purchased from Shanghai Sipper-BK Lab Animal Co. Ltd. The rats were kept at constant conditions (24 ± 1°C and 60% humidity) with free tap water and rodent food under a 12-hour light/dark cycle (lights on from 08:30 to 20:30. All experimental procedures were approved by the Experimental Animal Center of Nanjing University of Chinese Medicine (license No. SYXK (Su) 2014-0001). All applicable international, national, and institutional guidelines for the care and use of animals were followed.

The rats were allowed to acclimatize for a period of one week before being randomly assigned to five groups of eight. Group I: served as normal control (treated with intragastric administration of CMC-Na solution for 2 weeks); Group II: served as the model receiving alcohol (10 mL/kg/body weight, daily); Group III: received silymarin—is also the positive control group (200 mg/kg body weight, daily, dissolved in CMC-Na); Groups IV and V: received L-SC and H-SC extractum, respectively (4.0 g/kg and 8.0 g/kg body weight, daily, dissolved in CMC-Na); approximately the same volume of vehicle as the SC group was administered orally to the control and model groups.

### Analytical Bio-Sample Preparation

#### Sample Collection

The experiment continued for 2 weeks. At the end of treatment, rats were executed, and blood samples were drawn by cardiac puncture with heparinized tubes. Bile was collected from all of the rats through bile duct drainage by intubation in the 12 hours after drug administration. All bile samples were stored frozen at −80°C for UHPLC-Q/TOF-MS analysis. Blood (blood samples were obtained from the abdominal aorta) and liver tissues were collected quickly. Finally, liver tissues were placed in ice-cold 0.9% sodium chloride solution, perfused with physiological saline solution to remove blood cells, blotted on filter paper, and stored at −80°C for later use.

#### Sample Preparation

Each rat’s blood was centrifuged at 3600 rpm and 4°C for 10 min, and then the supernatant was stored at −80 °C and thawed prior to analysis. A total of 200 μL of the supernatant was added to cold methanol (600 μL), and the mixture then was shaken vigorously for 1 min. After centrifugation at 14,000 rpm for 10 min at 4 °C, the supernatant was concentrated to dryness before being reconstituted in 100 μL of the initial mobile phase. It was then centrifuged at 12,000 rpm for 5 min at 4 °C before UHPLC-Q/TOF-MS analysis.

Fresh bile samples were immediately centrifuged at 3600 rpm for 10 min at 4 °C to remove particle contaminants, and the supernatants were stored at −80 °C until UHPLC-Q/TOF-MS analysis.

### Histological Evaluations and Measurement of Serum and Liver Enzyme Activities

The histological changes in liver were assessed as described in Liu et al. (2013). The extent of hepatic damage was evaluated on H&E slides. The histological changes were scored according to the following criteria: 0, absent; 1, mild; 2, moderate; 3, severe; and 4, irreversible. The activities of ALT, AST, AKP, γ-GT/GGT, ROS, and MDA in serum were estimated spectrophotometrically using commercial diagnostic kits.

### UHPLC-Q/TOF-MS Analysis and Processing

UHPLC-Q/TOF-MS analysis was carried out on an Ekspert 100-XL UPLC system coupled to a hybrid quadrupole time-of-flight tandem mass spectrometer, which was equipped with Turbo V sources and a Turbo ion spray interface (AB Sciex Triple TOF 5600+). Chromatographic separation was performed on a C_18_ reversed-phase LC column (Agilent ZorBax SB-C_18_ 50 mm × 2.1 mm, 1.8 μm), and the column temperature was maintained at 25°C. The mobile phase consisted of 0.1% (v/v) formic acid water (solvent A), and an acetonitrile (solvent B) using a gradient program as follows. Plasma: linear gradient from 15% to 25% B (0 to 3 minutes); 25% to 35% B (5 to 7 minutes); 35% to 40% B (7 to 8 minutes); 40% to 45% B (8 to 11.5 minutes); 45% to 55% B (11.5 to 13 minutes); 55% to 65% B (13 to 14.5 minutes); 65% to 75% B (14.5 to 19 minutes); 75% to 100% B (19 to 24 minutes); isocratic 100% B for 1 minutes; back to 15% B within 2 minutes. Bile: linear gradient from 15% to 21% B (0 to 2 minutes); 21% B (2 to 4 minutes); 21% to 23% B (4 to 5 minutes); 23% B (5 to 8 minutes); 23% to 30% B (8 to 9 minutes); 30% B (9 to 13 minutes); 30% to 37% B (13 to 16 minutes); 37% B (16 to 20 minutes); 37% to 100% B (20 to 25 minutes); isocratic 100% B for 1 minutes; back to 15% B within 2 minutes. The flow rate was 0.3 mL/min, and the injection volume was 1 µL.

In this study, for the mass detection, the instrument was operated in positive and negative ion electrospray mode. The conditions of the MS/MS detector were as follows: ion spray voltage, 5000 V/-4500 V; turbo spray temperature, 550°C; decluttering potential (DP), 60 V/-55 V; collision energy (CE), 45 eV/-40 eV. Nitrogen was then used as a nebulizer and auxiliary gas, with the nebulizer (gas 1), heater (gas 2), and set curtain gases at 55, 55, and 35 psi, respectively. A full scan was run in both positive and negative modes (mass range: *m/z* 100 to 1500 amu; accumulation time: 200 ms). The qualitative analysis in this research adopted a critical parameter, which was collision energy spread (CES); this can increase sensitivity and reduce the potential for missing pieces of information. We set the CES at 15 eV/-20eV. The data were acquired using Analyst^®^ TF 1.6 software (AB Sciex).

### Multivariate Statistical Data Processing

The UHPLC-Q/TOF-MS data of all of the studied samples were analyzed using MarkerView and PeakView software (AB Sciex, Massachusetts, USA) for the purpose of identifying potential biomarkers for the discernment and quality control of the SC. In regard to the data collection, the method parameters were set as follows: retention time range from 0.5 to 20 minutes; mass range from 50 to 1500 Da; retention time tolerance 0.01 minute; mass tolerance 0.01 Da. For the peak integration, the peak width at a 5% height was one second, peak-to-peak baseline noise was 0.1, and the peak intensity threshold was 10. The resulting three-dimensional data, which were comprised of the peak number (*t*_R_-*m/z* pair), sample name, and ion intensity, were analyzed using a principal component analysis (PCA), accompanied by an orthogonal partial least squared discriminant analysis (OPLS-DA) within the Simca-P 14.0 software which was utilized in this study.

In the OPLS-DA modeling, the samples in different groups were classified by S-plots, and endogenous metabolites were distinguished according to the importance of different classifications in the projection (VIP) values. This indicated the significance of each variate to the classification. For measuring the quality of clustering within a PLS-DA scores plot, the degree-of-class-separation (DCS) was calculated between three groups, which was defined as the class-to-class variance divided by the sum of the within class variance on a scores plot using two sample types in the training set (Yao et al., 2014).

### Biomarker Identification and Metabolic Pathway Analysis

To identify potential biomarkers, we searched the METLIN database (https://www.sisweb.com/software/ms/wiley-metlin.htm), HMDB database (http://www.hmdb.ca/), and KEGG (http://www.genome.jp/kegg/) using the exact masses of potential biomarkers. The number of candidate molecules was reduced with the help of the retention times and typical MS/MS fragments and patterns. In this study, we finally compared the secondary fragment ion information of potential differential biomarkers with the standard secondary mass spectrometry results (under the same experimental conditions) in the HMDB and Massbank databases. We assume that when the degree of match is higher than 70%, they are the same substance. To entirely and visually illustrate the connections and differences among different groups, the relative intensities of the 41 differential metabolites in plasma and bile were displayed in a heat map generated by HCE 3.0 software.

### Network Target and Pathway Prediction of the Main Components in SC

According to the analytical results for the chemical constituents (show in **Figure 1**), the 10 primary compounds in SC were selected as the biological targets. The Canonical SMILES of 10 components were put into the SwissTargetPrediction database (http://www.swisstargetprediction.ch/) to obtain the Uniprot ID of the predicted targets. The Uniprot ID was then imported into the Kyoto Encyclopedia of Genes and Genomes (KEGG) database (http://www.genome.jp/kegg/) to predict the related pathways. The biological targets related to ALI were selected from the GAD database (https://geneticassociationdb.nih.gov/). The components–targets–pathways network was then established by using Cytoscape 3.6.1 software.

## Results

### SC Alleviated Alcohol-Induced Histology Changes in the Liver

**Figure 2:**
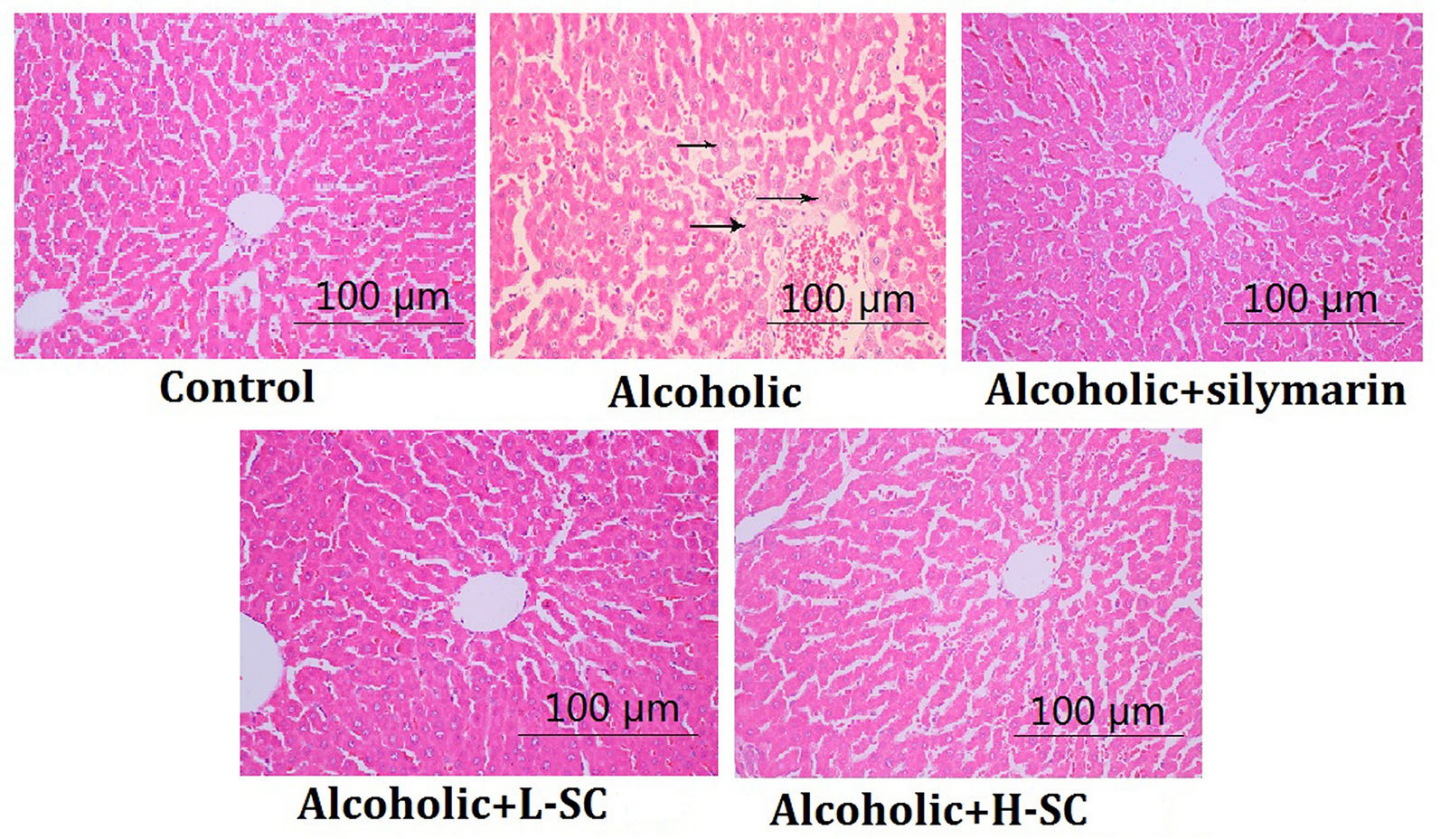
Morphological and histological evaluation of rat liver. Original magnification, 100 μm.

As shown in **Figure 2**, alcohol treatment caused several visible histological liver changes, including fatty degeneration, necrosis of focal hepatocytes, and infiltration of inflammatory cells. Moreover, the score for liver injury was evidently higher in the alcohol-treated rat than in controls (*P* < 0.01). However, L-SC and H-SC treatment alleviated the alcohol-induced damage in rat livers, and the H-SC treatment alleviated the alcohol-induced damage in rat livers more significantly than was seen in the L-SC group.

### SC Protects Against Alcohol-Induced Hepatic Dysfunction

**Figure 3:**
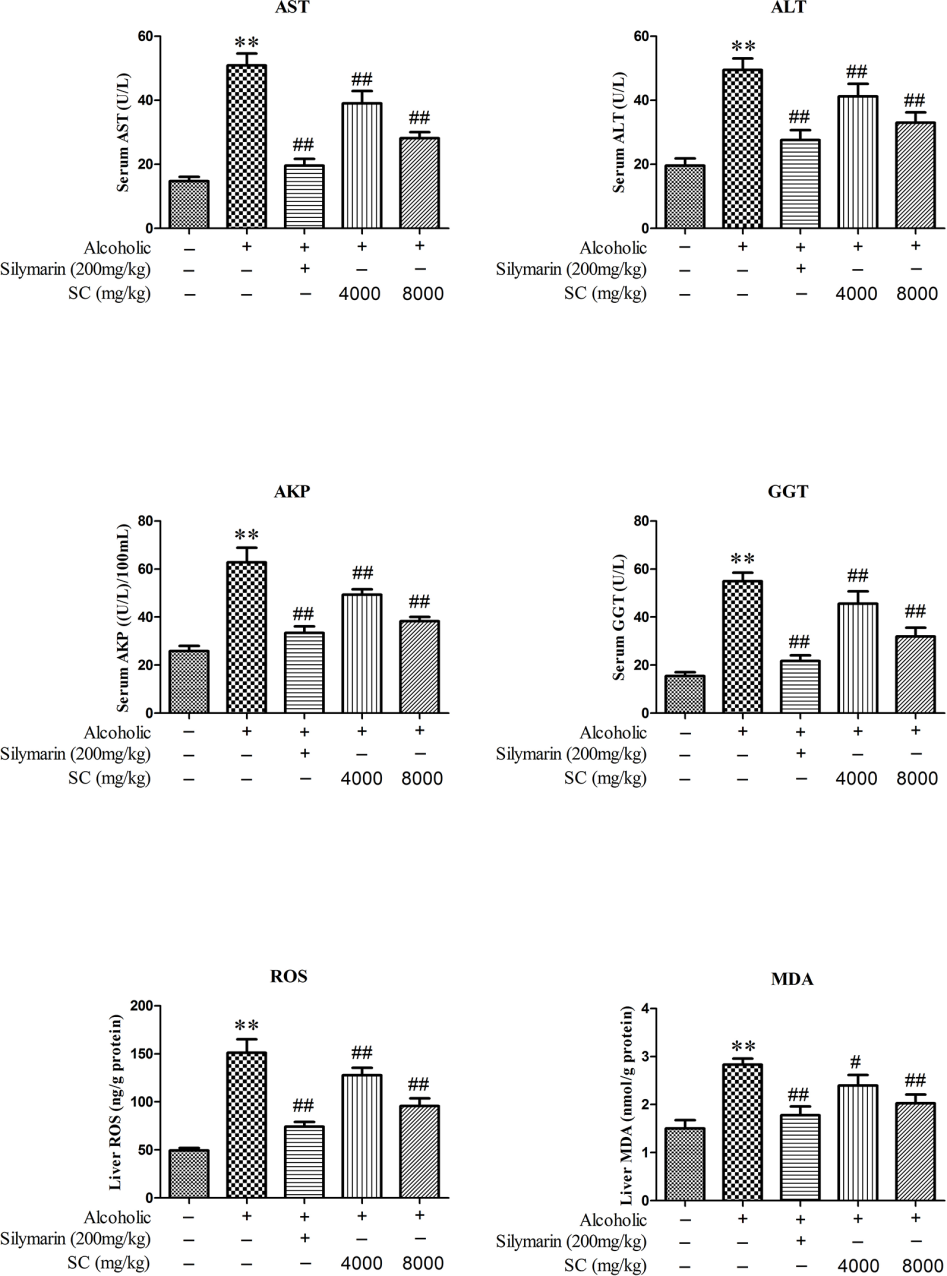
Effect of SC on alcohol-induced changes in hepatic functional markers. All values are expressed as mean ± SD (n = 8). ^**^P < 0.01, compared with the control group; ^##^ *P* < 0.01, ^#^ *P* < 0.05, *vs.* the alcohol-treated group.

In order to determine whether SC can reduce the liver damage in alcohol-treated rats, we measured the activities of ALT, AST, AKP, GGT, ROS, and MDA in serum (**Figure 3**). In alcohol-treated rats (the Model group), the activities of ALT, AST, AKP, GGT, ROS, and MDA in serum markedly increased as compared to in the normal controls (P < 0.01). Interestingly, the activity of L-SC/H-SC in the serum of alcohol-treated rats was significantly decreased (P < 0.01).

### Chromatographic Conditions and Q/TOF-MS Method Development

The plasma and bile samples were tested with different types of mobile phases, such as 0.05% and 0.1% formic and acetic acid in water, acetonitrile, and methanol. The best resolution and peak shapes were obtained in a mixture of methanol and aqueous 0.1% formic acid solution. To gather more information, multivariate statistical analysis was performed with both the positive and negative ion pattern monitoring data (Guo et al., 2014). The conditions of urine and serum samples were set respectively as 750 and 650 L/h of the desolvation gas flow and 500 and 550°C of the temperature. Superfluous solvent arising from a flow rate of 0.3 mL/min to the mass spectrometer was removed. Representative TIC chromatograms of the rat plasma and bile were obtained in the positive and negative ESI modes. (**Supplementary-1 Data, Figure S1**).

### Hepatoprotective Effects of SC Based on Metabolite Profiling

**Figure 4:**
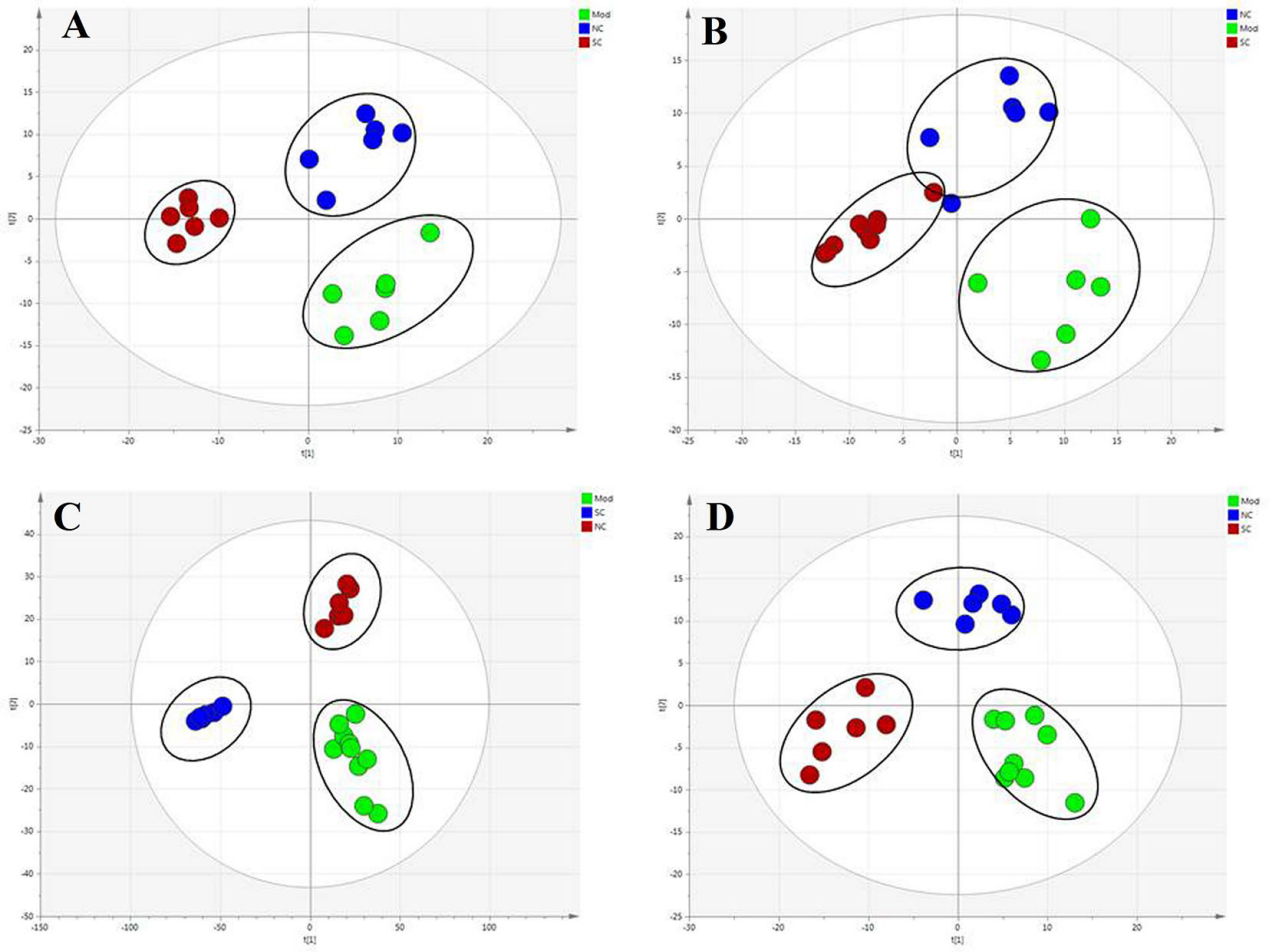
Score plot (t_1_/t_2_) of PLS-DA. **(A)** Plasma-ESI+, *R^2^X* = 0.567, *R^2^Y* = 0.995, *Q^2^Y* = 0.849; **(B)** plasma-ESI-, *R^2^X* = 0.570, *R^2^Y* = 0.992, *Q^2^Y* = 0.885; **(C)** bile-ESI+, *R^2^X* = 0.59381, *R^2^Y* = 0.991, *Q^2^Y* = 0.935; **(D)** bile-ESI-, *R^2^X* = 0.529, *R^2^Y* = 0.988, *Q^2^Y* = 0.838. Axes represent score t, one vector for each model dimension, new variables computed as linear combinations of the X’s. They provide a summary of X that best approximates the variation of both approximate X and predicted Y.

To compare the hepatoprotective effect of SC (in metabolomics research, we choose the H-SC with a strong pharmacodynamic effect for analysis), we further subjected the liver metabolic profiles of four groups of rats to multivariate analysis. PLS-DA was used to study the metabolic changes and appraise the curative effect of SC on rats. Samples from different groups and ions indicated to be responsible for the discrimination can be discriminated by PLS-DA, a public method for handling metabolomics data. As shown in **Figure 4**, the normal controls and model group were given two doses each, suggesting that they manifested significant liver protection. The hepatic protective effect stayed the same with the above plasma and bile biochemistry parameters and liver histopathology.

### Identification of Perturbed Biomarkers

**Figure 5:**
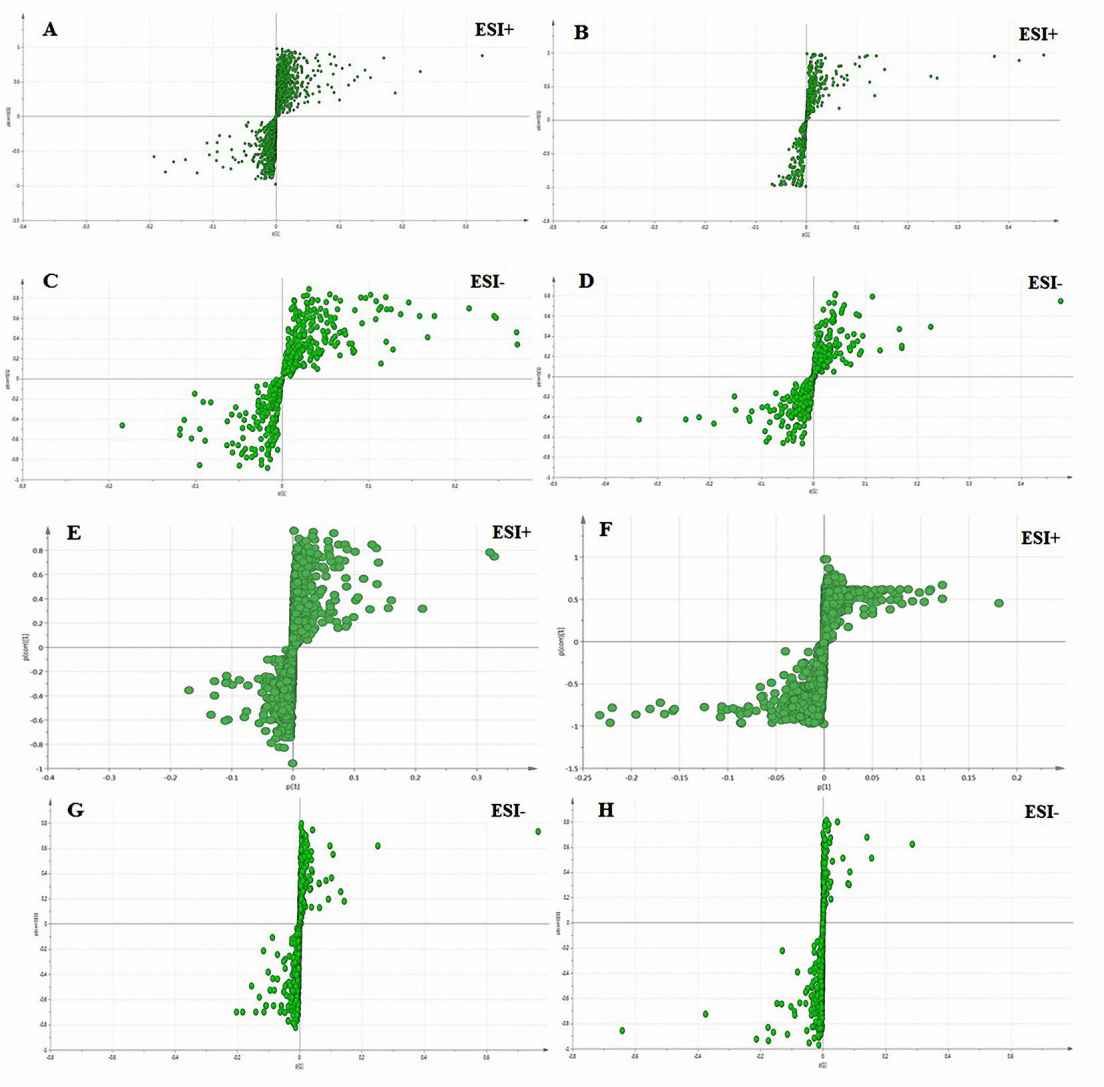
S-plots derived from the OPLS-DA used to select plasma **(A**–**D)** and bile **(E**–**H)** biomarkers: variables farthest from the origin were the most likely potential biomarkers due to their high contributions and correlations. **A**, **C**, **E**, and **G**: the model group was compared with the normal control group; **B**, **D**, **F**, and **H**: the SC-treated group was compared with the model group.

The variables (metabolites) that obviously lead to discrimination and clustering are identified according to pattern recognition and discriminant analysis, and then ANOVA is conducted. These differential metabolites were confirmed by the use of a separate sample t-test (spss19.0) in order to select the potential biomarkers that were worth studying (Xing et al., 2017). The critical p-value for significantly differential variables in the research was set at 0.05. Due to the observed intra-group variation, the supervised method, OPLS-DA (with a CV-ANOVA p-value less than 0.05) was further adopted to identify the variables responsible for separating the control group from the model group (**Figure 5**). The VIP value displayed the sequence of metabolites depending upon their key impact on clusters. First, a total of 88 and 64 metabolites with significant differences (P < 0.05, VIP > 1.0) between control and model groups were screened in plasma and bile samples, respectively (**Supplementary-1 Data, Tables S2–S5**).

Consequently, 21 significantly differential metabolites from plasma and 20 significantly differential metabolites from bile samples were selected for further study. The identification of these metabolites was then conducted as follows; the results of these metabolites are listed in **Tables 1** and **2**. PeakView software was used to generate the possible elements of the selected compound. The structure of potential biomarkers was illustrated by high-resolution MS and MS/MS spectra and various databases, including KEGG (http://www.genome.jp) and HMDB (http://www.hmdb.ca). Accordingly, the 21 metabolites from the plasma samples and the 20 metabolites from the bile samples were identified according to an elemental composition match, in the context of retention time, with the available databases. All of them were further confirmed, including retention times and MS/MS fragmentation patterns, by comparison with authentic standards.

**Table 1:** Identification of potential biomarkers in rat plasma.

No.	*t*_R_/min	Molecular ion *m/z*	Adduct	Metabolite	Formula	Error (ppm)	MS^2^ fragments	HMDB ID	Metabolic pathway
M_1_	15.29	758.5713	M+H	PE(15:0/22:2(13Z,16Z))	C_42_H_80_NO_8_P	2	703, 552, 432, 319, 263	HMDB0008909	I
M_2_	15.29	782.5718	M+H	No Hits	C_44_H_80_NO_8_P	-	-	-	No Hits
M_3_	15.30	806.5693	M+H	PC(20:5(5Z,8Z,11Z,14Z,17Z)/18:1(9Z))	C_46_H_80_NO_8_P	0	782, 786, 758, 703	HMDB0008498	I
M_4_	25.12	758.5700	M+H	PC(20:1(11Z)/14:1(9Z))	C_42_H_80_NO_8_P	1	703, 552, 432, 319, 263	HMDB0008296	I
M_5_	25.22	780.5563	M+H	PC(16:1(9Z)/20:4(5Z,8Z,11Z,14Z))	C_44_H_78_NO_8_P	3	758, 703, 478, 398, 214	HMDB0008015	I
M_6_	25.15	759.5763	M+IsoProp+Na+H	PE (14:0/P-18:0)	C_37_H_74_NO_7_P	1	734, 725, 703	HMDB0008851	I
M_7_	7.01	432.2809	M+2Na+H	1’-O-Acetylpaxilline	C_29_H_35_NO_5_	-	-	-	No Hits
M_8_	25.24	806.5694	M+CH_3_OH+H	PE(22:6(4Z,7Z,10Z,13Z,16Z,19Z)/P-18:1(9Z))	C_45_H_76_NO_7_P	0	782, 786, 758, 703	HMDB0009711	I
M_9_	25.21	828.5517	M+IsoProp+Na+H	PG(16:0/18:3(6Z,9Z,12Z))	C_40_H_73_O_10_P	3	743, 279, 253	HMDB0010576	I
M_10_	25.22	804.5538	M+H	PC(18:3(9Z,12Z,15Z)/20:4(5Z,8Z,11Z,14Z))	C_46_H_78_NO_8_P	1	788, 504, 301, 279, 257	HMDB0008180	I
M_11_	5.66	318.3011	M+H	Phytosphingosine	C_18_H_39_NO_3_	3	283, 282, 241, 239, 227, 209, 197, 183, 169	HMDB0004610	II
M_12_	5.83	362.3274	M+CH_3_OH+H	2-Palmitoyl Serinol	C_19_H_39_NO_3_	3	213, 209, 187, 183, 169, 155, 153, 141, 127	HMDB0013654	No Hits
M_13_	13.83	802.5558	M+Cl	PC(o-16:0/20:4(8Z,11Z,14Z,17Z))	C_44_H_82_NO_7_P	4	663, 569, 455, 439, 393, 377	HMDB0013407	I
M_14_	12.21	766.5337	M+Na-2H	PE(14:0/22:1(13Z))	C_41_H_80_NO_8_P	4	744, 534, 516, 424, 406, 337	HMDB0008842	I
M_15_	12.55	852.5704	M+Cl	PC(22:5(4Z,7Z,10Z,13Z,16Z)/P-18:1(11Z))	C_48_H_84_NO_7_P	3	713, 495, 481, 401, 329, 327, 285	HMDB0008720	I
M_16_	9.57	552.3085	M-H	LysoPE(0:0/24:6(6Z,9Z,12Z,15Z,18Z,21Z))	C_29_H_48_NO_7_P	2	355, 337, 122, 80, 78, 64, 62	HMDB0011499	III
M_17_	9.79	803.5602	M+K-2H	TG(14:1(9Z)/14:1(9Z)/18:4(6Z,9Z,12Z,15Z))	C_49_H_82_O_6_	1	747, 595, 583, 527, 477, 331, 283, 275	HMDB0047902	IV
M_18_	16.99	579.3897	M+Hac-H	Cyclopassifloic acid B	C_31_H_52_O_6_	1	503, 489, 473, 459, 433, 401	HMDB0038388	No Hits
M_19_	25.61	850.5629	M-H	PE(DiMe(9,3)/DiMe(13,5))	C_47_H_82_NO_10_P	3	695, 429, 377, 333, 275, 249, 151, 140, 122, 78	HMDB0061500	I
M_20_	25.59	830.5872	M+Cl	PC(20:2(11Z,14Z)/P-18:1(11Z))	C_46_H_86_NO_7_P	4	691, 473, 459, 441, 379, 305, 291, 261	HMDB0008359	I
M_21_	25.63	826.5626	M+Hac-H	PE(22:4(7Z,10Z,13Z,16Z)/16:0)	C_43_H_78_NO_8_P	3	768, 750, 627, 512, 454, 389, 313	HMDB0009583	I

I, Glycerophospholipid metabolism; II, Sphingolipid metabolism; III, Inflammatory mediator regulation of TRP channels; IV, Fat degradation.

**Table 2:** Identification of potential biomarkers in rat bile.

No.	*t*_R_/min	Molecular ion *m/z*	Adduct	Metabolite	Formula	Error (ppm)	MS fragments	HMDB ID	Metabolic pathway
M_1_	2.2	141.0198	M+H+K	5-phosphonooxy-L-lysine	C_6_H_15_N_2_O_6_P	3	128, 114, 101, 99, 72, 68, 56, 44	HMDB0059600	i
M_2_	2.34	397.1530	M+2K-H	13-HETE	C_20_H_32_O_3_	3	259, 257, 245, 229, 189, 177, 149, 131	HMDB0012567	ii
M_3_	2.38	345.9906	2M+ACN+H	3-Sulfinylpyruvic acid	C_3_H_4_O_5_S	3	134, 116, 106, 90, 78, 43	HMDB0001405	ii
M_4_	4.35	369.1576	M+2K-H	3b,17a-Dihydroxy-5a-androstane	C_19_H_32_O_2_	5	277, 257, 247, 233, 217, 201, 177, 161, 135	HMDB0000412	iii
M_5_	4.78	429.2011	2M+Na	Tryptophanamide	C_11_H_13_N_3_O	0	187, 178, 169, 159,	HMDB0013318	iii
M_6_	6.47	445.1953	2M+K	L-Acetylcarnitine	C_9_H_17_NO_4_	1	204, 159, 149, 109, 96	HMDB0000201	ii
M_7_	7.73	408.2643	M+H+K	PG(18:0/18:1(9Z))	C_42_H_81_O_10_P	2	733, 719, 691, 677, 663, 605, 593, 479, 341, 325, 267, 225	HMDB0010604	iv
M_8_	13.48	561.3630	M+NH_4_	LysoPC(20:4(5Z,8Z,11Z,14Z))	C_28_H_50_NO_7_P	6	446, 432, 361, 347, 287, 269, 245, 217	HMDB0010395	iv
M_9_	12.60	514.2867	M-H	Taurohyocholate	C_26_H_45_NO_7_S	4	498, 406, 391, 363, 349, 166	HMDB0011637	iii
M_10_	12.26	496.2739	M-H_2_O-H	Taurocholic acid	C_26_H_45_NO_7_S	1	484, 482, 406, 391, 363, 347, 149, 124	HMDB0000036	iii
M_11_	2.46	646.9096	2M-H	Trichloroethanol glucuronide	C_8_H_11_Cl_3_O_7_	4	242, 240, 188, 186, 182, 175, 154, 152, 146, 144, 131, 117	HMDB0042049	iii
M_12_	9.69	512.2697	M-H	Sulfolithocholylglycine	C_26_H_43_NO_7_S	2	432, 416, 414, 386, 372, 359, 331, 315	HMDB0002639	iii
M_13_	9.99	1025.5408	M+Hac-H	PIP(20:3(5Z,8Z,11Z)/18:1(9Z))	C_47_H_84_O_16_P_2_	3	533, 499, 481, 463, 356, 320, 338, 211, 159, 126	HMDB0010013	iv
M_14_	14.75	530.2670	M+K-2H	LysoPC(16:1(9Z))	C_24_H_48_NO_7_P	3	501, 499, 498, 497, 464, 448, 415, 225	HMDB0010383	iv
M_15_	17.79	514.2717	M+Cl	LysoPE(18:1(11Z)/0:0)	C_23_H_46_NO_7_P	2	281, 263, 140, 122, 96, 82, 80, 78, 64, 62	HMDB0011506	iv
M_16_	11.40	510.2522	M-H_2_O-H	Glycochenodeoxycholate 3-sulfate	C_26_H_43_NO_8_S	1	448, 432, 430, 402, 388, 373, 347,116, 100, 84	HMDB0002497	iii
M_17_	13.25	517.2867	M+Na-2H	Postin	C_22_H_40_N_8_O_5_	0	479, 449, 425, 301, 295, 210, 198, 173, 167, 139	HMDB0005772	No Hits
M_18_	15.09	498.2891	M-H	Taurochenodesoxycholic acid	C_26_H_45_NO_6_S	1	481, 480, 330, 289, 288, 106, 96, 79	HMDB0000951	iii
M_19_	12.66	498.2888	M-H	Taurodeoxycholic acid	C_26_H_45_NO_6_S	1	482, 468, 390, 375, 347, 329, 149, 105, 76	HMDB0000874	iii
M_20_	5.86	254.0756	M+Na-2H	Glutaminylserine	C_8_H_15_N_3_O_5_	1	185, 171, 144, 130, 112, 84, 58, 43	HMDB0028806	i

i, Amino acid metabolism; ii, Fatty acid metabolism; iii, Bile acid metabolism; iv, Lipid metabolism.

As indicated in **Tables 1** and **2**, the model group was significantly different from the control group in terms of the 21 plasma metabolites and 20 bile metabolites. There were significant changes between the metabolites in the SC (H-SC) group and the model group. The related pathways of each biomarker were also listed by retrieving data from the KEGG PATHWAY Database (http://www.genome.jp/kegg/).

To achieve an overview of the allocations of different groups of potential biomarkers, we executed an OPLS-DA S-plot of the potential biomarkers. The purpose of the OPLS-DA S-plot is to effectively show the potential biomarkers in the distribution in different samples. These good indicators of the correlations are displayed by the arrows among the variables. Relative to the arrows, the location of the samples in different groups was a good indication that that variable had the greatest impact. These findings were consistent with **Tables 1** and **2**. As shown in the data in **Figure 5**, the plasma and bile data were perfectly divided into two groups.

**Figure 6:**
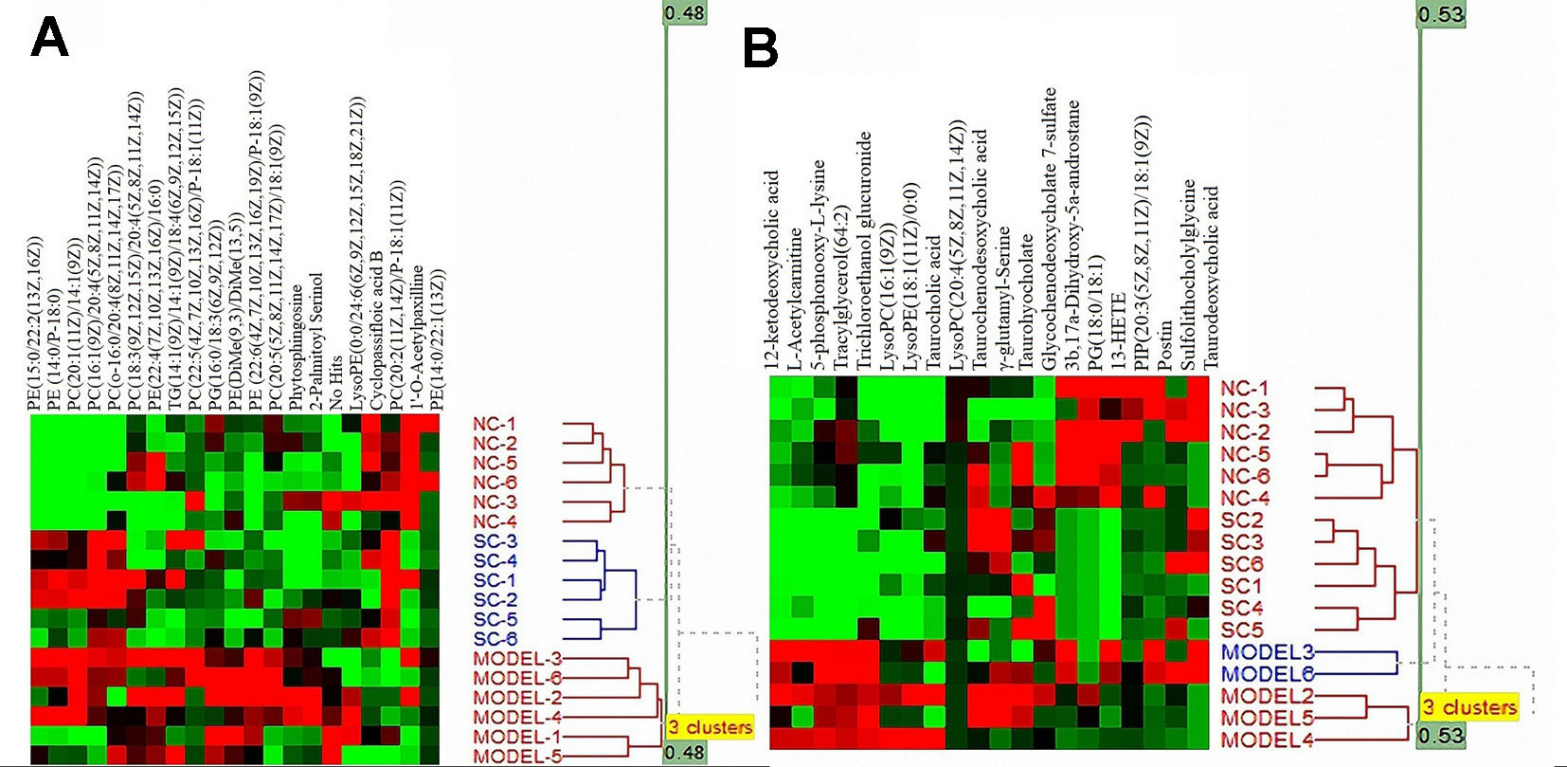
Heat maps of differential metabolites from plasma **(A)** and bile **(B)** samples.

The otherness potential of these metabolic biomarkers for ALI, especially between the model and SC groups, was evaluated in the validation data. As shown in **Figure 6**, the heat map of the 21 biomarkers in plasma (**Figure 6A**) and 20 biomarkers in bile (**Figure 6B**) demonstrated clear differential metabolic profiles between SC, model, and normal controls in the validation data.

### Metabolic Pathway Analysis

**Figure 7:**
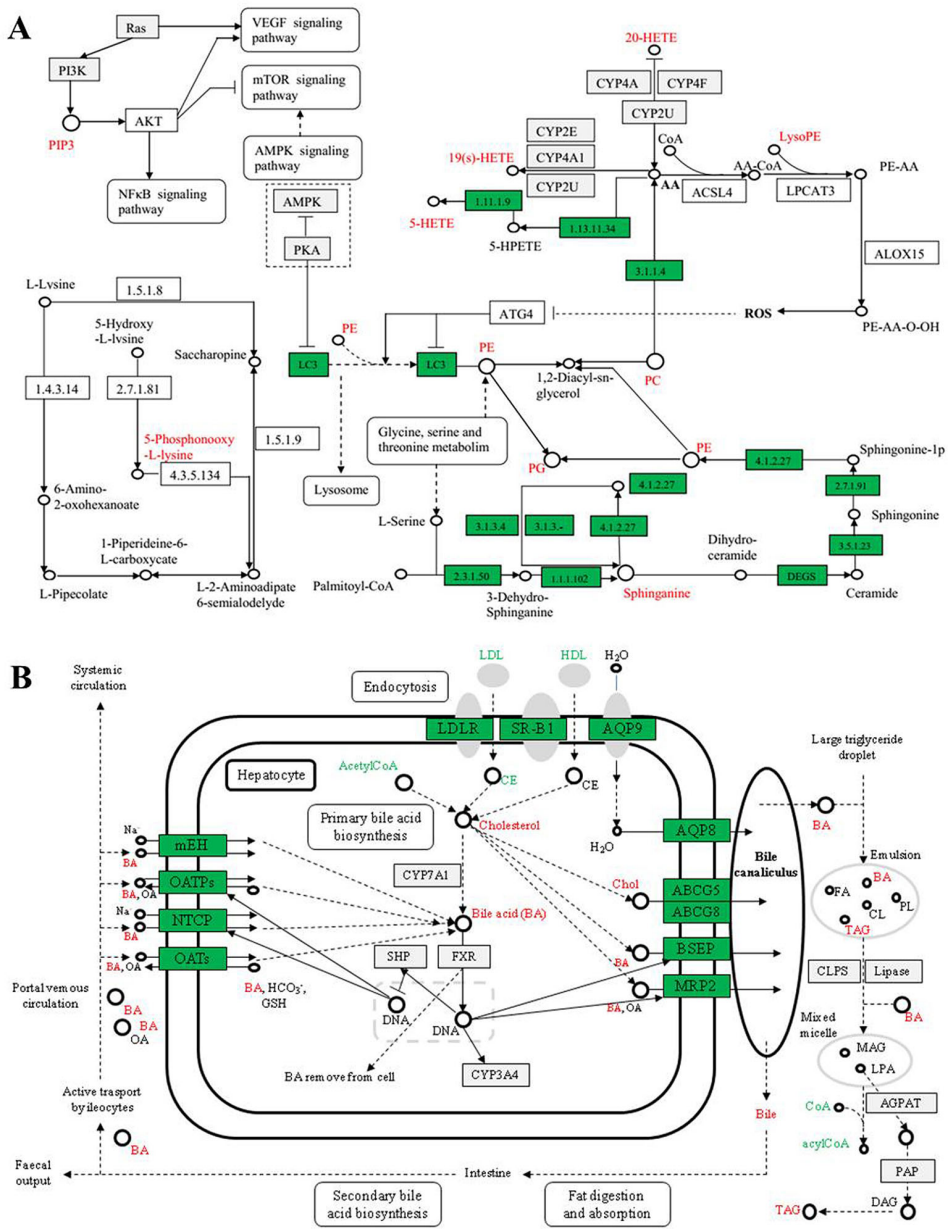
Network of the potential biomarkers in plasma **(A)** and bile **(B)** that changed between the ALI rats, normal controls, and SC-treated group. Red metabolite names indicate that they were detected in our study.

The variations of the 41 perturbed metabolites that differ between the SC and model groups in the alcoholic-treated rat livers were connected with multiple metabolic pathways, including glycerophospholipid metabolism, sphingolipid metabolism, inflammatory mediator regulation, bile acids metabolism, lipid metabolism, fatty acid oxidation, oxidative stress, nucleotide synthesis, and amino acid metabolisms, based on the KEGG database (http://www.genome.jp/kegg/pathway.html) results. The recovery trend of perturbed metabolites suggested that the mechanism of SC-induced protection from acute alcoholic liver injury in rats was relevant to the metabolic pathways, including glycerophospholipid metabolism, lipid metabolism, bile acid metabolism, amino acid metabolism, ketone body metabolism, and glutathione metabolism (**Figure 7**).

The results showed the metabolic networks of the potential biomarkers that changed in ALI in the normal controls, the model group, and SC on the basis of metabolic pathways. **Figure 7**, which shows the metabolic networks of the potential biomarkers that changed significantly for ALI between the normal control, model, and SC groups, indicates clear influences on the metabolic pathways, which could be put down to the fact that SC provided protection from acute alcoholic liver injury in rats.

### Lipid Metabolism

Due to liver injury, several kinds of metabolites involved in glycerophospholipid metabolism were derived from rats treated with alcohol in this research. These included phosphatidyl ethanolamine (PE), lysophosphatidyl ethanolamine (LysoPE), phosphatidylcholine (PC), lysophosphatidyl choline (LysoPC), and phosphatidyl glycerol (PG).

The main metabolic pathways involved in PE include biosynthesis of the glycosyl phosphatidylinositol (GPI) group, autophagy, retrograde endogenous cannabinoid signal, glycerol phospholipid metabolism, and biosynthesis of secondary metabolites, etc. LysoPE was originally found to be hemolytic but is now used to refer generally to the absence of phosphatidylcholine chains. LysoPE mainly involves inflammatory mediators with TRP channels regulating metabolic pathways. The channel can indirectly regulate inflammatory mediators in tissue damage, such as PGE2, bradykinin, ATP, nerve growth factor, and proinflammatory cytokine. The metabolic levels of the above metabolites were all abnormal in the model group, and intervention with SC showed a tendency to return to the normal state. It is suggested that the protective effect of SC on liver injury may be closely related to the regulation of phospholipid metabolism and inflammatory mediators.

The major metabolic pathways involved in PC and PG are glycerol phospholipid metabolism, alpha-linolenic acid metabolism, arachidonic acid metabolism, and choline metabolism in cancer. In addition, PC can also resist fatty liver, alcoholic liver, and other liver diseases and effectively prevent damage to and protect the liver. Cholinergic metabolism is considered to be a metabolic marker associated with tumorigenesis and tumor progression. With the activation of carcinogenic signaling pathways such as RAS conversion and the occurrence of the PI3K-AKT pathway, such as the overexpression of hypoxia-inducible factor-1 (HIF1)-related transcription factors and the activation the mediated cycle of cholinase, the choline level of the precursor and membrane phospholipid decomposition products is increased. In rats with liver injury, the disorder of the above metabolic pathway was improved to some extent after intervention with SC.

Sphingosine, also known as neuro sphingosine, is an eighteen-carbon amino alcohol-containing an unsaturated alkyl chain. It is a type of sphingolipid and is one of the important components of the cell membrane. Plant sphingosine is a metabolic intermediate of sphingosine-1-phosphoric acid, the sphingosine sphingosine sphingosine kinase-2, a metabolite of sphingosine as a metabolite of sphingolipid, is produced during the metabolic process of sphingosine. Recently, sphingosine-1-phosphoric acid has been proved to be effective in preventing liver and kidney injury (Park et al., 2010). In this research, the concentration of sphingosine decreased in the model group, indicating that CCl_4_ damage in rats may remove normal metabolic protection. Tetrahydrocorticosterone is a compound of steroid hormone biosynthesis. In addition, studies have shown that steroid hormones control lipid metabolism and neuro sphingolipids, including ceramide and sphingosine, which have been found to regulate the secretion of steroid hormones at multiple levels (Lucki and Sewer, 2010; Yao et al., 2014). Therefore, tetrahydrocorticosterone may indirectly protect the liver by affecting sphingosine-1-phosphorylation of steroid hormone biosynthesis. SC can regulate the metabolic level of sphingosine so that the concentration of tetrahydrocorticosterone is restored to normal, thus improving or protecting from rat liver injury.

### Bile Acid Metabolism

Bile acid is the final product of cholesterol metabolism in the body. Bile acids are a physiological cleanser that promotes the excretion, absorption, and transport of fat and sterols in the intestines and liver. Changes in the composition of abnormal bile acids are closely related to liver injury (Yao et al., 2014). The 12-ketodeoxycholic acid, 3b, 17a-Dihydroxy-5a-androstane, Taurohyocholate (a bile acid), Taurocholic acid, Taurodeoxycholic acid, bezoar deoxycholic acid, and Glycochenodeoxycholate 7-sulfate concentrations in the bile of model group rats compared with the control group have different degrees of up-regulation or down-regulation, suggesting bile acid metabolism disturbance in the alcoholic hepatic injury rat model, causing lipid metabolism dysfunction. Due to liver-function damage, the secretion of bilirubin is increased, the synthesis of primary bile acids by hepatocytes is increased, and the amount of 12-ketoxy cholic acid and 3b, 17a-Dihydroxy-5a-androstane excreted is increased, which causes it to increase in bile. As a result, the more serious the liver damage is, the higher they are in the bile (Adachi et al., 1988).

Compared with the model group, the levels of 12-keto deoxycholic acid and 3b, 17a-Dihydroxy-5a-androstane in the SC group were significantly decreased, suggesting that SC can regulate bile acid metabolism and have a lipid-lowering effect when used as an intervention in liver injury. Taurocholic acid, taurocholocholic acid, and Niuhuang goose deoxycholic acid are bile salts formed by cholic acid, deoxycholic acid, or goose deoxycholic acid and taurine, usually sodium salt. Taurohyocholate is a metabolite that is catalyzed by the CYP3A4 enzyme. The content of taurocholic acid and taurocholic acid in liver-injury rats increased, and the content of taurohyocholate decreased significantly. Therefore, we speculated that the metabolism of taurocholic acid and taurocholic acid decreased; that is, the activity of CYP3A4 might be inhibited. The metabolic levels of the three metabolites were improved in the SC group. Therefore, it is possible to speculate that SC can regulate the metabolism of bile acid by inducing the activity of the CYP3A4 enzyme. From the experimental results, the content of 7-thiocarbamate-deoxycholic acid in the model group and SC group increased significantly, while the SC-intervention group significantly decreased their metabolic abnormalities. From this point of view, the anti-liver injury effect of SC is also related to the regulation of oxidative stress and lipid peroxidation.

### Amino Acid Metabolism

The results showed that the concentration of 5-phosphonyl-L-lysine in bile increased significantly in liver-injury rats, indicating that the ALI model is closely related to amino acid metabolism disorder. 5-phosphonyl-L-lysine is the substrate of hydroxyl lysine kinase and 5-phospho hydroxyl-L-lysine phosphatase, while hydroxyl lysine kinase and 5-phospho hydroxyl-L-lysine phosphatase are necessary enzymes for regulating lysine phosphorylation and controlling cell growth and differentiation. Lysine is the essential amino acid of the human body. It is known as the first essential amino acid of the human body. Under normal circumstances, it acts as an amino acid to synthesize various proteins or participate in the regulation of protein synthesis. With the occurrence of liver injury or disease, the liver function disorder of amino acid metabolism in the liver cells decreases, leading to concentration of some amino acids in the blood (bile, lysine, tyrosine, and tryptophan), and decreased amino acid uptake capacity in the body itself (Rosen et al., 1977; Morgan et al., 1982). The level of 5-phosphoryl-L-lysine in the SC group was significantly lower than that in the model group, indicating that SC could significantly regulate the amino acid metabolism and intervened in liver injury.

### Fatty Acid Metabolism

13-Hydroxyeicosatetraenoic acid (13-HETE) is one of the main metabolites of 15-lipoxygenase (15-LOXs) and is the endogenous ligand of PPARR. It is also a metabolite of arachidic acid (AA) in rat liver particles and is a red algae formed by the hydroxylation of AA in many cell types (Yuan et al., 2000). There are three different enzymes involved in HETE synthesis: lipoxygenase (LOX), prostaglandin H (PGH), and cytochrome P450 enzyme. Therefore, the metabolic level of 13-HETE can reflect the metabolic level of AA, CYP450, PGH, and LOXs in the body. Tracylglycerol (64:2) is an important triglyceride, the main component of extremely low-density lipoprotein (VLDL) and chylous particles, and plays an important role in energy metabolism and dietary fat transport. L-Acetylcarnitine (ALC) promotes acetyl coenzyme A into the mammalian mitochondrial matrix to oxidize fatty acids. In addition to its metabolism, ALC has a unique neuroprotective, neuromodulation, and neurotrophic effect, which may play an important role in fighting a variety of diseases. Compared with the model group, the level of 13-HETE was increased by the SC group. The content of TG in the SC group was significantly lower than that in the model group, suggesting that SC can also protect the liver by decreasing the content of TG (Duan et al., 2010). Compared with the model group, the content of L-Acetylcarnitine in the SC group was decreased, and it was presumed that it could protect the liver by maintaining the oxidation–antioxidant balance of the body.

### Component–Target–Pathway Network Analysis

**Figure 8:**
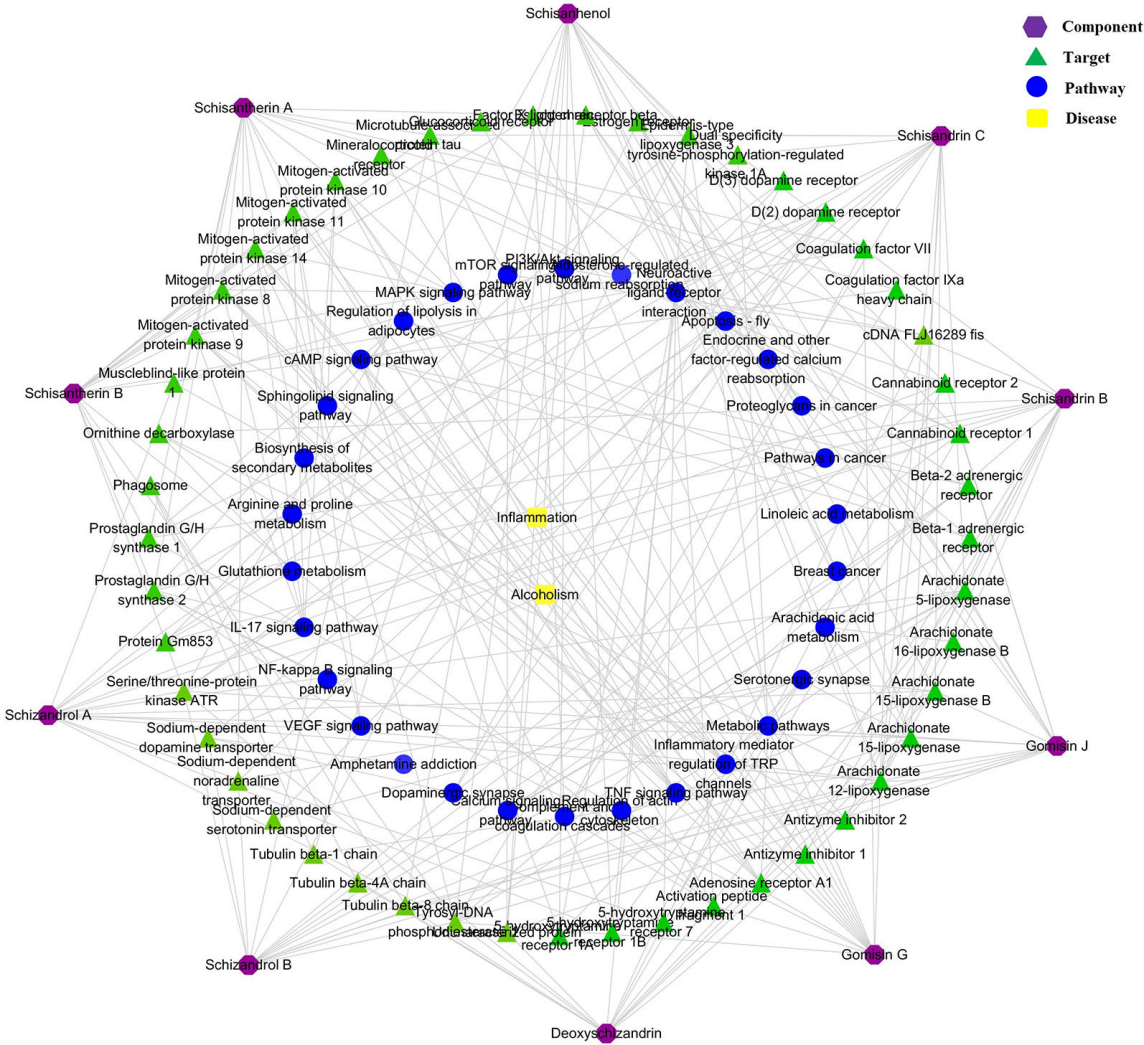
Compound–target–metabolic pathway network.

The component–target–pathway network results are shown in **Figure 8**. The network predicted that 150 targets were closely related to the 10 chemical components. A total of 111 metabolic pathways were related to the 150 targets, in which five metabolic pathways, including glycerophospholipid metabolism, sphingolipid metabolism, four arachidonic acid metabolism, bile acid metabolism, and amino acid metabolism, were most consistent with the metabolomics results. Thus, the scientific reliability of the results of metabolomics analysis are verified *via* another aspect.

## Discussion

The authors suggest that the mechanism of Schisandra chinensis in liver is multifaceted, multi-channel, and multi-target, mainly including PC, PE, metabolites of LysoPE, Sphingonine, HETEs, PIP, and bile acid; the main metabolic pathways include glycerophospholipid metabolism, sphingolipid metabolism, four arachidonic acid metabolism, bile acid metabolism, amino acid metabolism, and cell autophagy pathways, including PI3K-Akt, mTOR, AMPK, NF-kappa B; the main metabolic enzymes include ROS, CYP450, CoA, Acetyl-CoA, etc. A literature search indicates that experimental research has described the possible pathogenesis of alcoholic liver injury (oxidative stress and lipid peroxidation, infiltration of inflammatory cytokines and endotoxin) in detail, and the results confirmed these conclusions from many aspects. Intervention with drugs (extract of SC) after liver injury in rats altered metabolism by affecting the expression level of related enzymes and signaling factors to regulate a series of endogenous metabolites.

### Metabolic Pathways of AA and Glycerol-Phospholipids

The metabolism of glycerol is closely related to the metabolism of arachidonic acid through the bridge of PC (phosphatidylcholine), such as HETEs, PE, PC, PG, LysoPE, and CYP450 enzymes. The experimental results showed that in the lipid metabolism in rats with alcoholic liver injury serious disorder, the vast majority of lipids (triglycerides, phospholipids, cholesterol, and steroids) showed high expression, and with the drug intervention (SC), the lipid metabolism level returned to the normal state. It is suggested that the lipid metabolism level of liver-injury rats is seriously unbalanced, and the internal mechanism of the hepatoprotective effect of SC is closely related to it.

### ROS and CYP450 Enzymes

Ethanol produces ROS by CYP2E1 metabolism, leading to oxidative damage. In addition, long-term ethanol exposure can damage mitochondrial function by causing structural changes in mitochondria and inhibition of oxidative phosphorylation and the three tricarboxylic acid (TCA) cycle, resulting in a reduction in bio-energy, increased ROS, decreased GSH, damage to mitochondrial DNA, and inhibition of protein synthesis. The increases in the permeability sensitivity of mitochondria through necrosis and the cell apoptosis pathway induced by liver injury ROS promote lipid peroxidation and damage to the cell cytoplasm and cell membrane. Analysis of the related metabolic pathway map, which showed direct relations to the generation of the LysoPE, ROS, and AA metabolic pathways, indicates that many CYP450 enzymes (such as CYP2E, CYP2U, CYP4A) are involved in the formation of ROS. In the experiment, the LysoPE of the model group was significantly up-regulated, and it was significantly lower after treatment with SC than in the model group (P < 0.01). That is to say, SC can better inhibit the production of ROS, so as to effectively improve alcohol-induced liver injury.

### PI3K/Akt/mTOR Signaling Pathway

PI3K is an oncogene composed of two subunits that regulate subunit p85 and catalytic subunit P110, mainly in cytoplasm. Akt, also known as protein kinase B (PKB), is an evolutionarily conserved serine/threonine protein kinase that mainly exists in cytoplasm and is a downstream molecule of B (PI3K). The mTOR (mammalian target of rapamycin) is the target of rapamycin downstream of Akt-signaling molecules. Research shows that the PI3K/Akt activation of the mTOR signaling pathway can inhibit the apoptosis of cells, and the activation of PI3K can cause mutations in the normal Akt physiological function of the cells, and generate induced tumors, to suppresses tumor cell apoptosis (Guertin and Sabatini, 2005; Banumathy and Cairns, 2010). Liver injury is a necessary stage for the pathological prophase of many liver diseases. In this study, the expression of PIP in alcoholic liver-injury rats was high, and it was speculated that the upstream PI3K gene and downstream Akt protein kinase also showed a high expression trend. Therefore, the PI3K/Akt/mTOR signaling pathway is highly activated during liver injury, leading to the aggravation of liver injury. However, the results of this study showed that PIP was effectively inhibited after treatment with SC and speculated that the mechanism of hepatoprotective effect of SC was closely related to the PI3K/Akt/mTOR signaling pathway.

## Conclusion

Although used for thousands of years, the exact anti-ALI mechanism of SC remains poorly understood. We attempted to find the key molecules involved in the pharmacological process of SC to potentially explain its complete and complex biochemical mechanisms. In this study, a method that combined UHPLC-Q/TOF-MS-based metabolomics and network pharmacology revealed that SC-induced biomarkers change with ALI in rat coronary artery ligation model, which has never reported before. The results of this study showed that the protective effect of SC in liver was related to energy metabolism, lipid metabolism, ketone body metabolism, glutathione metabolism, and amino acids metabolism. In our previous study, the protective effect of SC on the liver could not be clearly distinguished by the serum biochemistry parameters and liver histopathology results. Therefore, where the traditional index is not sufficiently sensitive, the LC-MS/MS-based metabolomics approach can be used as a sensitive method to compare and appraise drug indication.

## Data Availability Statement

All datasets generated for this study are included in the article/**Supplementary Material**.

## Ethics Statement

This study was carried out in accordance with the principles of the Basel Declaration and the recommendations of all applicable international, national, and institutional guidelines for the care and use of animals. The protocol was approved by the Experimental Animal Center of Nanjing University of Chinese Medicine (license no. SYXK (Su) 2014-0001).

## Author Contributions

Conceptualization: LS and TL. Investigation: JM and MH. Data curation: DJ and CM. Writing—original draft preparation: LS and CF. Writing—review and editing: TL and HT. Supervision: CM. Project administration: TL. Language revision: LS and CF.

## Funding

This research study was supported by the National Natural Science Fund of China (No. 81373971, 81873008).

## Conflict of Interest

The authors declare that the research was conducted in the absence of any commercial or financial relationships that could be construed as a potential conflict of interest.
