# Supplementary material for: Integrated Plasma and Bile Metabolomics Based on an UHPLC-Q/TOF-MS and Network Pharmacology Approach to Explore the Potential Mechanism of Schisandra chinensis-Protection From Acute Alcoholic Liver Injury
Source: Front Pharmacol. 2020 Jan 16;10:1543. doi: 10.3389/fphar.2019.01543 (PMC6975200; doi:10.3389/fphar.2019.01543)
Supplement: Supplementary file 1 [file DataSheet_1.pdf]

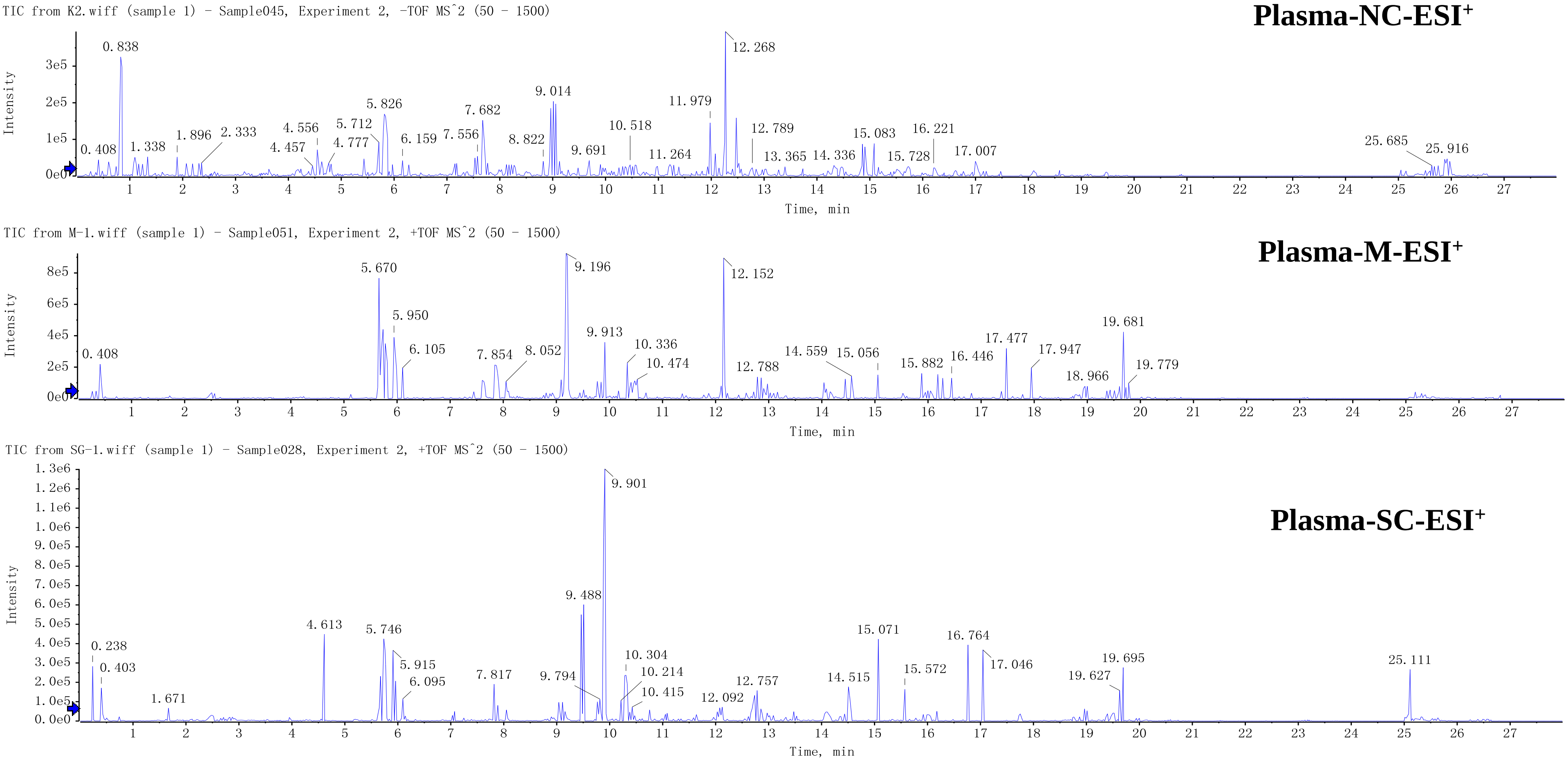

TIC from K2.wiff (sample 1) - Sample045, Experiment 2, -TOF MS^2 (50 - 1500)
Plasma-NC-ESI+
Intensity
Time, min
0.838
12.268
9.014
5.826
7.682
11.979
10.518
12.789
16.221
15.083
2.333
1.896
4.556
5.712
4.777
4.457
1.338
0.408
6.159
7.556
8.822
9.691
11.264
13.365
14.336
15.728
17.007
25.685
25.916
TIC from M-1.wiff (sample 1) - Sample051, Experiment 2, +TOF MS^2 (50 - 1500)
Plasma-M-ESI+
Intensity
Time, min
5.670
9.196
12.152
5.950
9.913
10.336
10.474
6.105
0.408
7.854
8.052
12.788
14.559
15.056
15.882
16.446
17.477
17.947
18.966
19.681
19.779
TIC from SG-1.wiff (sample 1) - Sample028, Experiment 2, +TOF MS^2 (50 - 1500)
Plasma-SC-ESI+
Intensity
Time, min
9.901
9.488
4.613
5.746
0.238
0.403
5.915
6.095
1.671
7.817
9.794
10.304
10.214
10.415
12.092
12.757
14.515
15.071
15.572
16.764
17.046
19.627
19.695
25.111

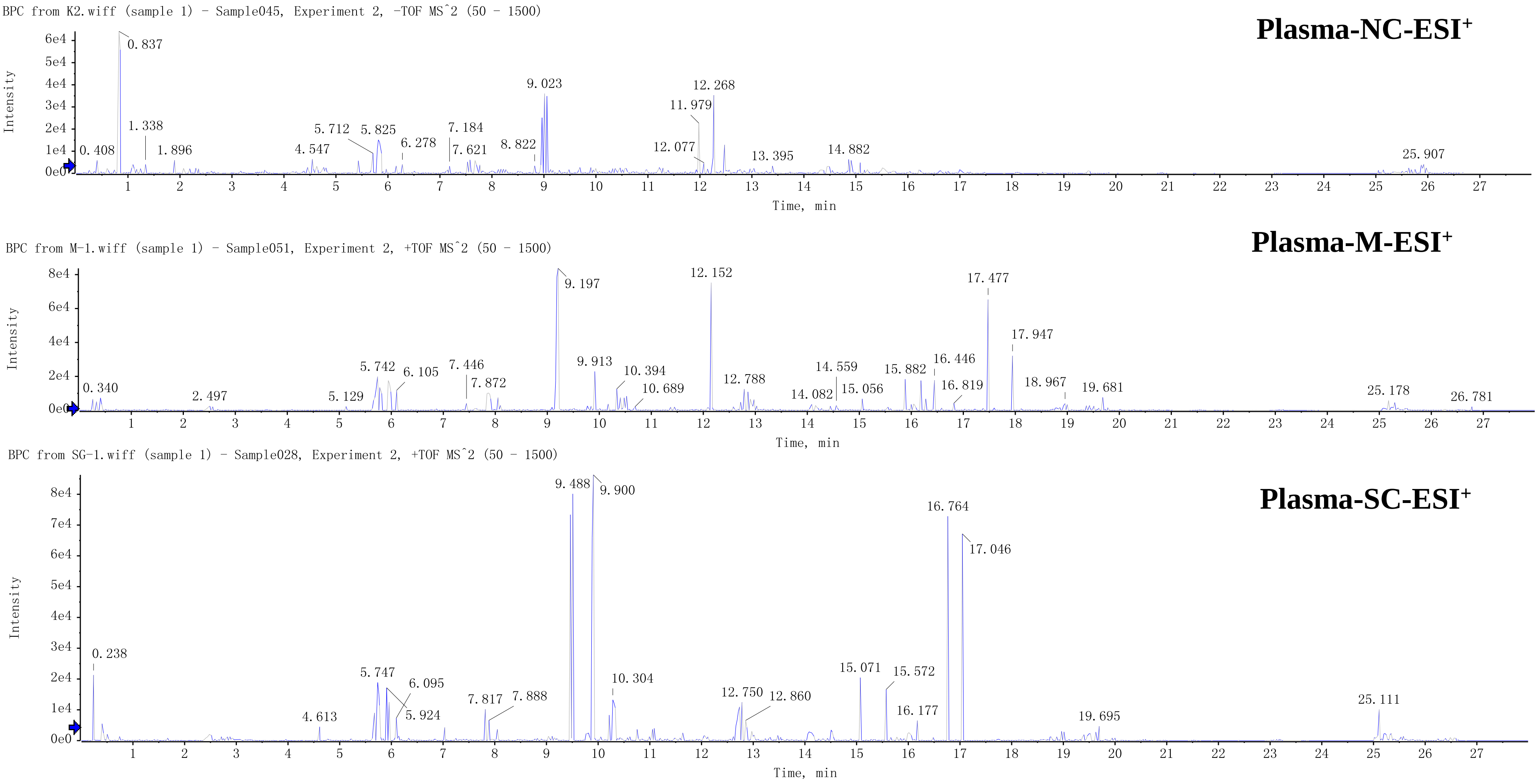
BPC from K2.wiff (sample 1) - Sample045, Experiment 2, -TOF MS^2 (50 - 1500)
Plasma-NC-ESI+
Intensity
6e4
5e4
4e4
3e4
2e4
1e4
0e0
0.837
0.408
1.338
1.896
4.547
5.712
5.825
6.278
7.184
7.621
8.822
9.023
11.979
12.268
12.077
13.395
14.882
25.907
Time, min
BPC from M-1.wiff (sample 1) - Sample051, Experiment 2, +TOF MS^2 (50 - 1500)
Plasma-M-ESI+
Intensity
8e4
6e4
4e4
2e4
0e0
0.340
2.497
5.129
5.742
6.105
7.446
7.872
9.197
9.913
10.394
10.689
12.152
12.788
14.082
14.559
15.056
15.882
16.446
16.819
17.477
17.947
18.967
19.681
25.178
26.781
Time, min
BPC from SG-1.wiff (sample 1) - Sample028, Experiment 2, +TOF MS^2 (50 - 1500)
Plasma-SC-ESI+
Intensity
8e4
7e4
6e4
5e4
4e4
3e4
2e4
1e4
0e0
0.238
4.613
5.747
6.095
5.924
7.817
7.888
9.488
9.900
10.304
12.750
12.860
15.071
15.572
16.177
16.764
17.046
19.695
25.111
Time, min

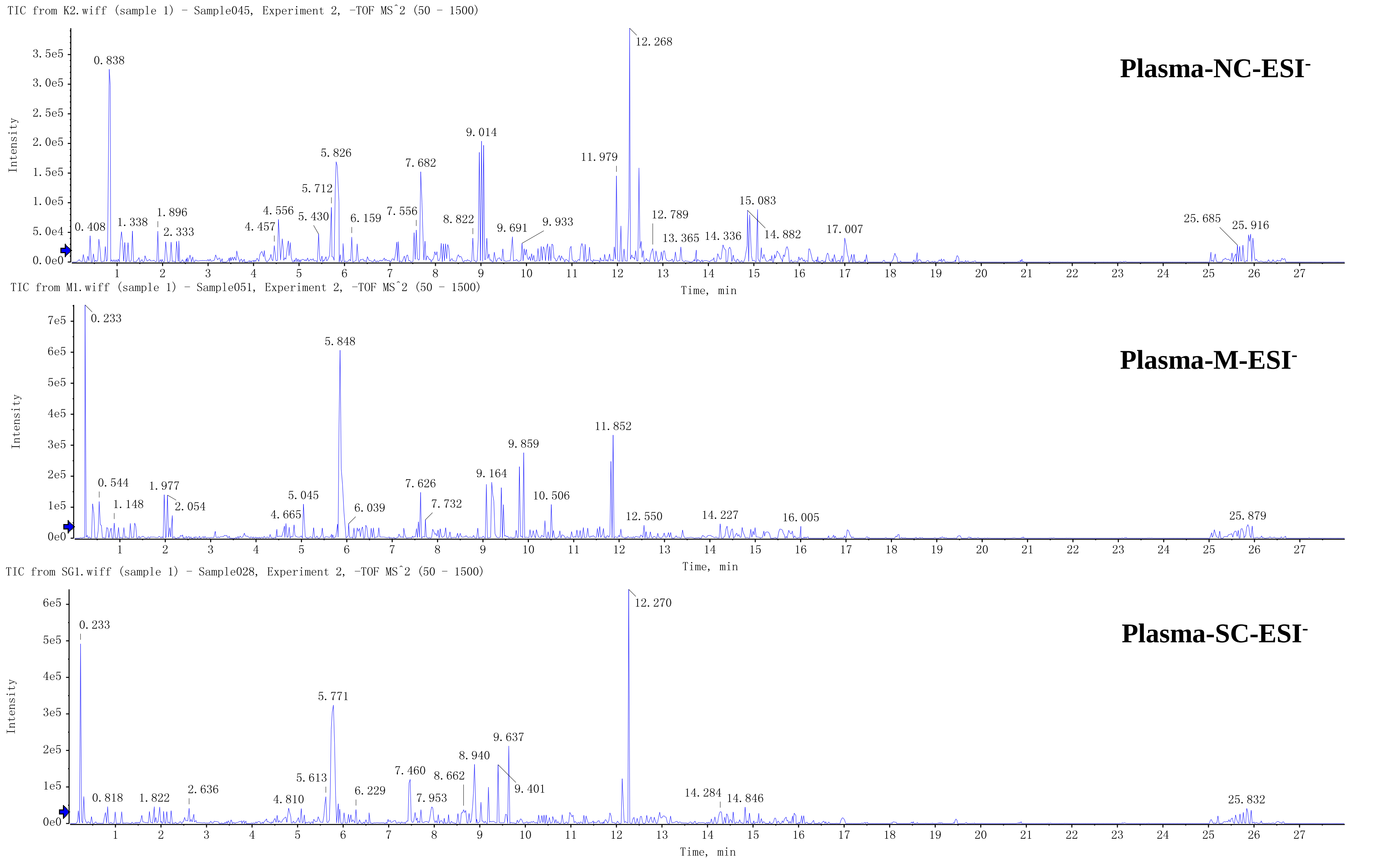
TIC from K2.wiff (sample 1) - Sample045, Experiment 2, -TOF MS^2 (50 - 1500)
Plasma-NC-ESI-
Intensity
Time, min
0.408
0.838
1.338
1.896
2.333
4.457
4.556
5.430
5.712
5.826
6.159
7.556
7.682
8.822
9.014
9.691
9.933
11.979
12.268
12.789
13.365
14.336
14.882
15.083
17.007
25.685
25.916
TIC from M1.wiff (sample 1) - Sample051, Experiment 2, -TOF MS^2 (50 - 1500)
Plasma-M-ESI-
Intensity
Time, min
0.233
0.544
1.148
1.977
2.054
4.665
5.045
5.848
6.039
7.626
7.732
9.164
9.859
10.506
11.852
12.550
14.227
16.005
25.879
TIC from SG1.wiff (sample 1) - Sample028, Experiment 2, -TOF MS^2 (50 - 1500)
Plasma-SC-ESI-
Intensity
Time, min
0.233
0.818
1.822
2.636
4.810
5.613
5.771
6.229
7.460
7.953
8.662
8.940
9.401
9.637
12.270
14.284
14.846
25.832

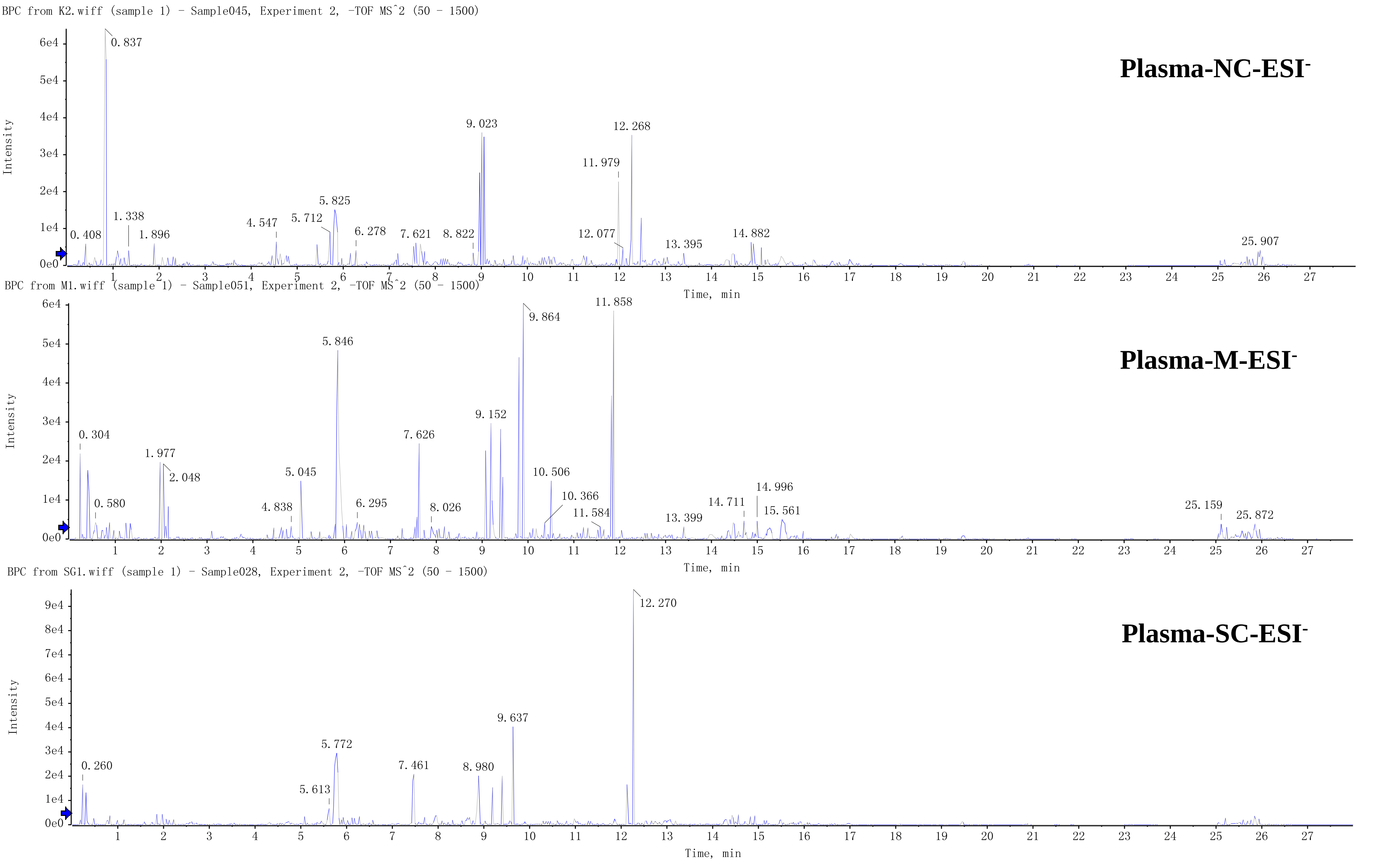
BPC from K2.wiff (sample 1) - Sample045, Experiment 2, -TOF MS^2 (50 - 1500)
Plasma-NC-ESI-
Intensity
0.837
9.023
12.268
11.979
5.825
1.338
4.547
5.712
6.278
7.621
8.822
12.077
0.408
1.896
13.395
14.882
25.907
Time, min
BPC from M1.wiff (sample 1) - Sample051, Experiment 2, -TOF MS^2 (50 - 1500)
Plasma-M-ESI-
Intensity
11.858
9.864
5.846
9.152
0.304
7.626
1.977
2.048
5.045
10.506
10.366
11.584
0.580
4.838
6.295
8.026
14.711
14.996
15.561
13.399
25.159
25.872
Time, min
BPC from SG1.wiff (sample 1) - Sample028, Experiment 2, -TOF MS^2 (50 - 1500)
Plasma-SC-ESI-
Intensity
12.270
9.637
5.772
0.260
7.461
8.980
5.613
Time, min

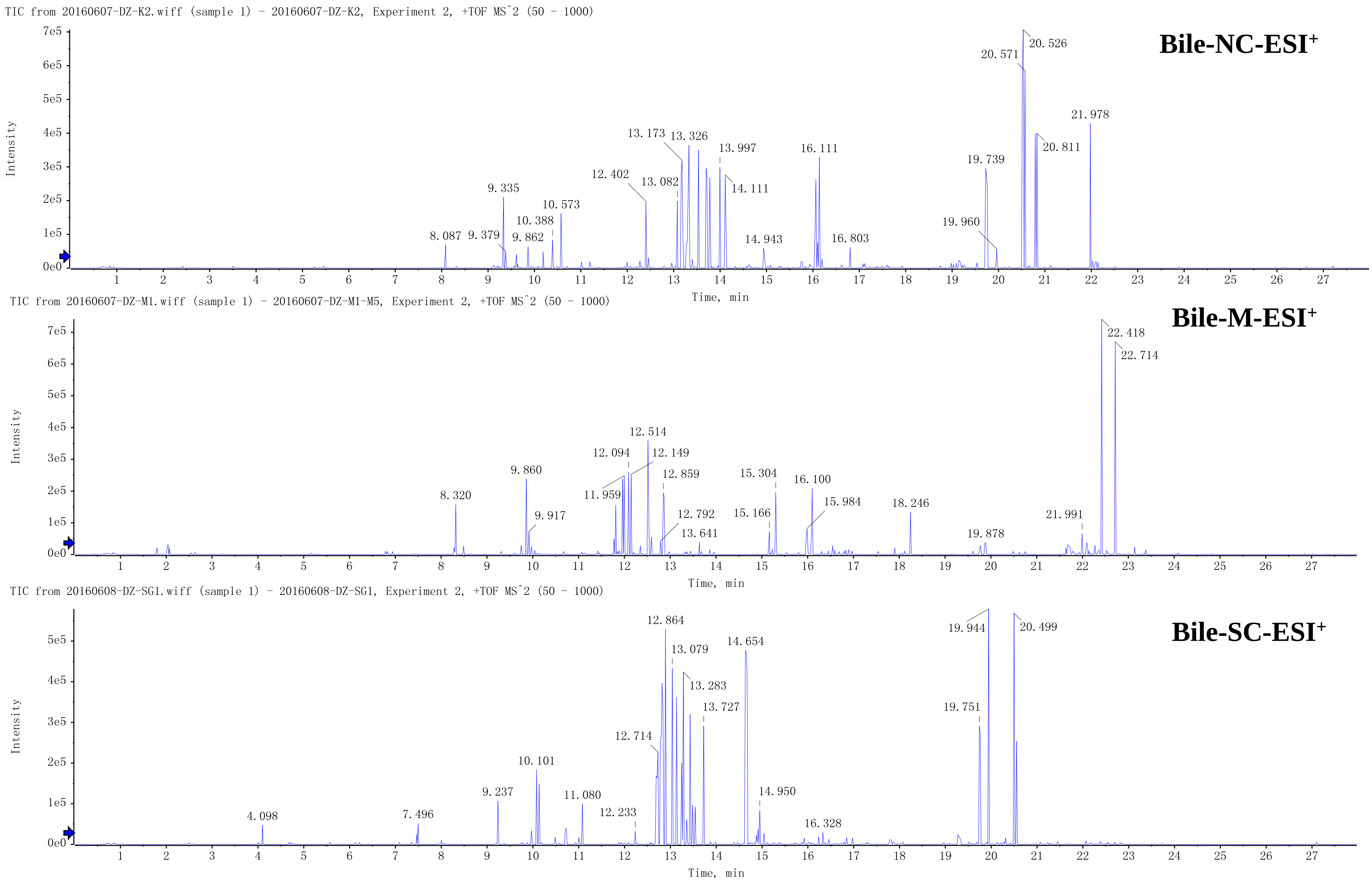
TIC from 20160607-DZ-K2.wiff (sample 1) - 20160607-DZ-K2, Experiment 2, +TOF MS^2 (50 - 1000)
Bile-NC-ESI+
20.526
20.571
21.978
13.173
13.326
13.997
16.111
20.811
19.739
12.402
13.082
14.111
9.335
10.573
10.388
8.087
9.379
9.862
14.943
16.803
19.960
Intensity
Time, min
TIC from 20160607-DZ-M1.wiff (sample 1) - 20160607-DZ-M1-M5, Experiment 2, +TOF MS^2 (50 - 1000)
Bile-M-ESI+
22.418
22.714
12.514
12.094
12.149
9.860
12.859
15.304
16.100
8.320
11.959
15.984
18.246
9.917
12.792
15.166
21.991
13.641
19.878
Intensity
Time, min
TIC from 20160608-DZ-SG1.wiff (sample 1) - 20160608-DZ-SG1, Experiment 2, +TOF MS^2 (50 - 1000)
Bile-SC-ESI+
12.864
19.944
20.499
13.079
14.654
13.283
13.727
19.751
12.714
10.101
9.237
11.080
14.950
4.098
7.496
12.233
16.328
Intensity
Time, min

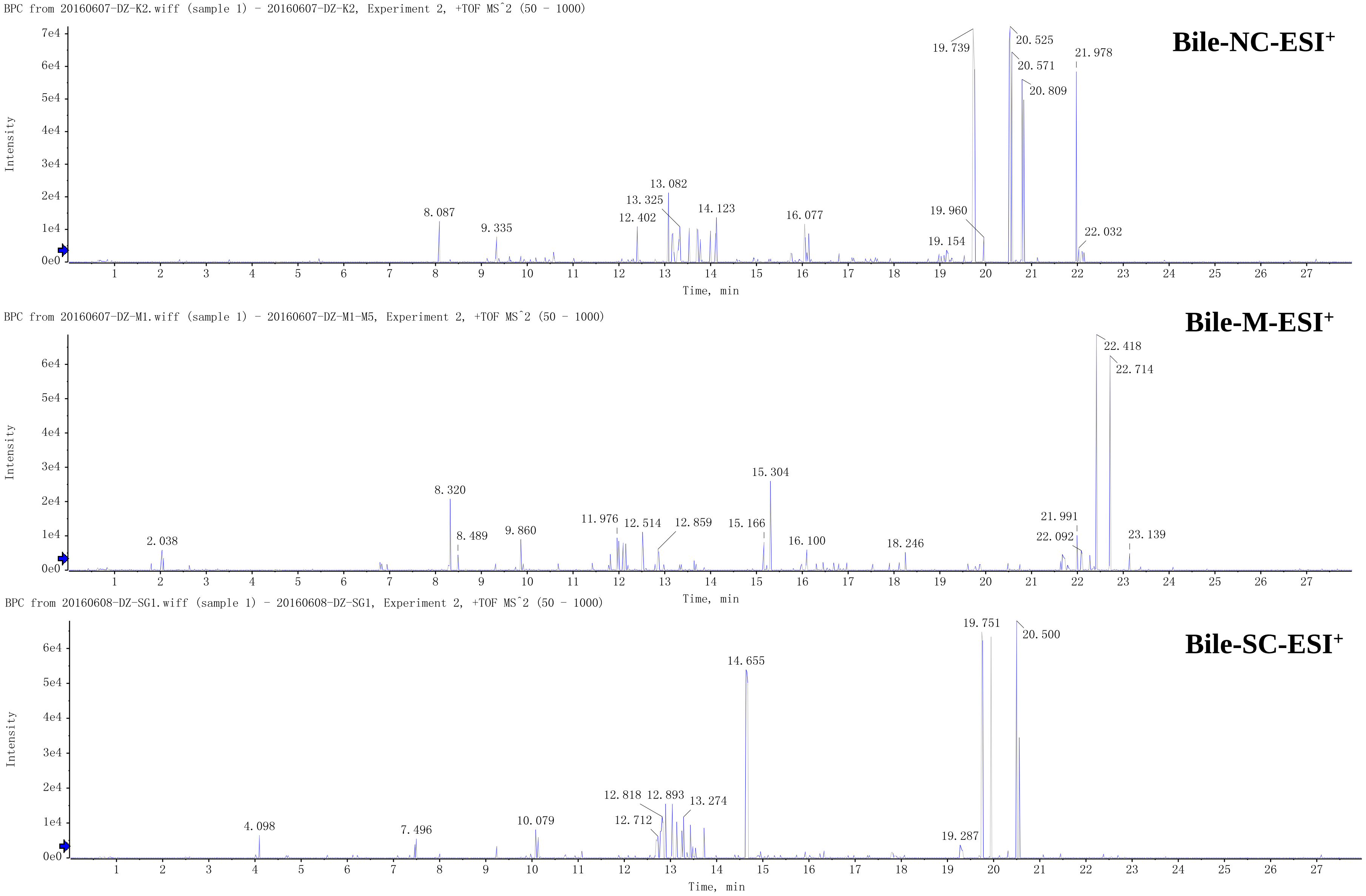
BPC from 20160607-DZ-K2.wiff (sample 1) - 20160607-DZ-K2, Experiment 2, +TOF MSˆ2 (50 - 1000)
Bile-NC-ESI+
Intensity
Time, min
8.087
9.335
12.402
13.325
13.082
14.123
16.077
19.154
19.960
19.739
20.525
20.571
20.809
21.978
22.032
BPC from 20160607-DZ-M1.wiff (sample 1) - 20160607-DZ-M1-M5, Experiment 2, +TOF MSˆ2 (50 - 1000)
Bile-M-ESI+
Intensity
Time, min
2.038
8.320
8.489
9.860
11.976
12.514
12.859
15.166
15.304
16.100
18.246
21.991
22.092
22.418
22.714
23.139
BPC from 20160608-DZ-SG1.wiff (sample 1) - 20160608-DZ-SG1, Experiment 2, +TOF MSˆ2 (50 - 1000)
Bile-SC-ESI+
Intensity
Time, min
4.098
7.496
10.079
12.712
12.818
12.893
13.274
14.655
19.287
19.751
20.500

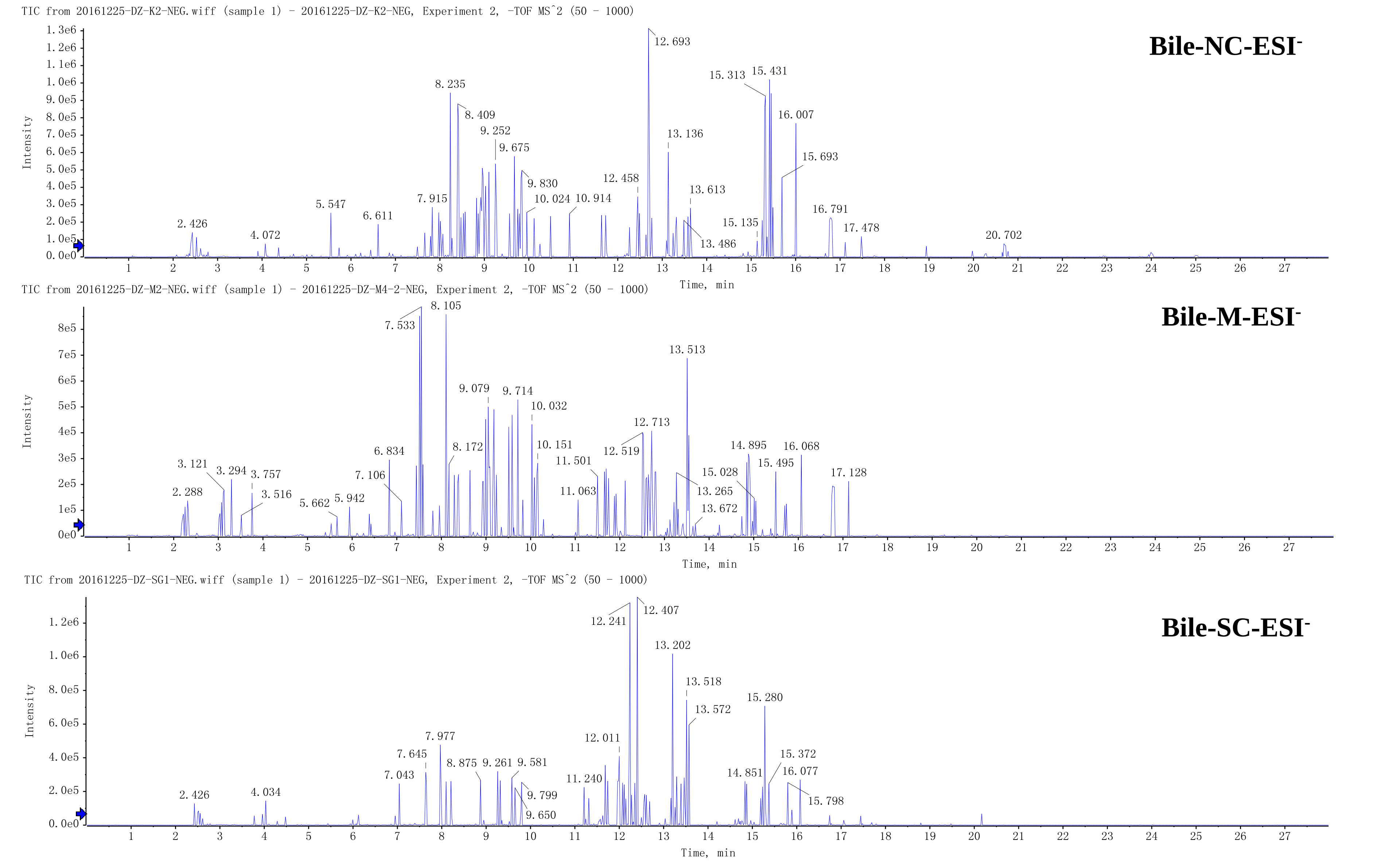
TIC from 20161225-DZ-K2-NEG.wiff (sample 1) - 20161225-DZ-K2-NEG, Experiment 2, -TOF MS^2 (50 - 1000)
Bile-NC-ESI-
Intensity
Time, min
2.426
4.072
5.547
6.611
7.915
8.235
8.409
9.252
9.675
9.830
10.024
10.914
12.458
12.693
13.136
13.613
13.486
15.135
15.313
15.431
16.007
15.693
16.791
17.478
20.702
TIC from 20161225-DZ-M2-NEG.wiff (sample 1) - 20161225-DZ-M4-2-NEG, Experiment 2, -TOF MS^2 (50 - 1000)
Bile-M-ESI-
Intensity
Time, min
2.288
3.121
3.294
3.757
3.516
5.662
5.942
6.834
7.106
7.533
8.105
8.172
9.079
9.714
10.032
10.151
11.063
11.501
12.519
12.713
13.513
13.265
13.672
14.895
15.028
15.495
16.068
17.128
TIC from 20161225-DZ-SG1-NEG.wiff (sample 1) - 20161225-DZ-SG1-NEG, Experiment 2, -TOF MS^2 (50 - 1000)
Bile-SC-ESI-
Intensity
Time, min
2.426
4.034
7.043
7.645
7.977
8.875
9.261
9.581
9.799
9.650
11.240
12.011
12.241
12.407
13.202
13.518
13.572
14.851
15.280
15.372
16.077
15.798

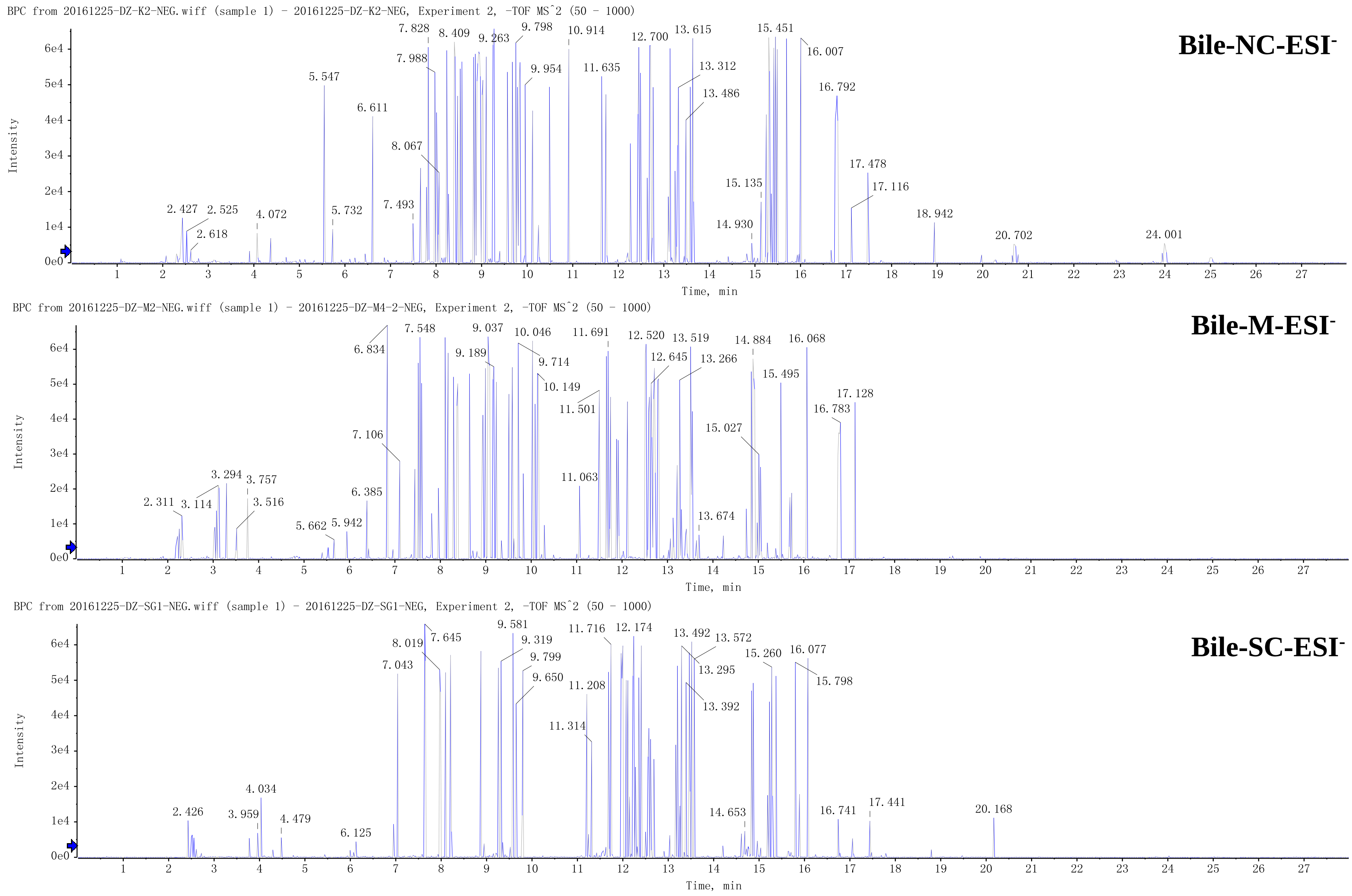

BPC from 20161225-DZ-K2-NEG.wiff (sample 1) - 20161225-DZ-K2-NEG, Experiment 2, -TOF MSˆ2 (50 - 1000)
Bile-NC-ESI-
Intensity
Time, min
2.427
2.525
2.618
4.072
5.547
5.732
6.611
7.493
7.828
7.988
8.067
8.409
9.263
9.798
9.954
10.914
11.635
12.700
13.615
13.312
13.486
14.930
15.135
15.451
16.007
16.792
17.478
17.116
18.942
20.702
24.001
BPC from 20161225-DZ-M2-NEG.wiff (sample 1) - 20161225-DZ-M4-2-NEG, Experiment 2, -TOF MSˆ2 (50 - 1000)
Bile-M-ESI-
Intensity
Time, min
2.311
3.114
3.294
3.757
3.516
5.662
5.942
6.385
6.834
7.106
7.548
9.037
9.189
10.046
9.714
10.149
11.501
11.063
11.691
12.520
12.645
13.519
13.266
13.674
14.884
15.495
15.027
16.068
16.783
17.128
BPC from 20161225-DZ-SG1-NEG.wiff (sample 1) - 20161225-DZ-SG1-NEG, Experiment 2, -TOF MSˆ2 (50 - 1000)
Bile-SC-ESI-
Intensity
Time, min
2.426
3.959
4.034
4.479
6.125
7.043
8.019
7.645
9.581
9.319
9.799
9.650
11.716
11.208
11.314
12.174
13.492
13.572
13.295
13.392
14.653
15.260
16.077
15.798
16.741
17.441
20.168
