# Supplementary material for: Integrated Plasma and Bile Metabolomics Based on an UHPLC-Q/TOF-MS and Network Pharmacology Approach to Explore the Potential Mechanism of Schisandra chinensis-Protection From Acute Alcoholic Liver Injury
Source: Front Pharmacol. 2020 Jan 16;10:1543. doi: 10.3389/fphar.2019.01543 (PMC6975200; doi:10.3389/fphar.2019.01543)
Supplement: Supplementary file 2 [file DataSheet_2.docx]

**1. The detailed methods of UHPLC-Q/TOF-MS**

*1.1 Liquid chromatography*

A UPLC-Q-TOF/MS analysis was conducted on an Agilent UPLC system coupled to a hybrid quadrupole time-of-flight tandem mass spectrometer, which was equipped with Turbo V sources, and a Turbo ion spray interface (AB Sciex Triple TOF 5600). A chromatographic separation was performed on a C_18_ reversed phase LC column (Agilent ZorBax SB-C_18_ 50 mm x 2.1 mm, 1.8 μm) with a Security Guard HPLC C_18_ column (4.6 mm, 0.2 μm), and the column temperature was maintained at 25◦C. The mobile phase consisted of 0.1% (v/v) formic acid water (solvent A), and an acetonitrile (solvent B) using a gradient program as follows: linear gradient from 25% to 40% B (0 to 2 minutes); 40% to 45% B (2 to 3 minutes); 45% to 55% B (3 to 8 minutes); 55% to 65% B (8 to 10 minutes); 65% to 75% B (10 to 15 minutes); 75% to 100% B (15 to 17 minutes); isocratic 100% B for 1 minutes; and then back to 25% B for 2 minutes. The flow rate was 0.3 ml/min, and the injection volume was 1 µL.

*1.2 Mass spectrometry*

In this study, for the mass detection, the instrument was operated in a positive ion electro spray mode. The conditions of the MS/MS detector were as follows: ion spray voltage, 5.5 kV; turbo spray temperature, 550◦C; declustering potential (DP), 60 V; and collision energy (CE), 35 eV. Then, nitrogen was used as a nebulizer and auxiliary gas, and the nebulizer (gas 1), heater (gas 2), and curtain gases were set to 55, 55, and 35 psi, respectively. A full scan was run in a positive mode, with a mass range from m/z 100 to 1000 amu, and with a 200 ms accumulation time. For the IDA criteria, the eight most intense fragment ions of each analyte that exceeded 50 cps were selected for the production scan, and the ion scan ranged from 50 to 1000 amu, with a 70 ms accumulation time. In this study, the collision energy spread (CES) was an important parameter in the qualitative analysis, which had the ability to improve the sensitivity and reduce the omissions of the fragment information. In this run, the CES was set at 10 eV, which enabled an average EPI scan spectrum to be obtained when the CE was 35, 45, and 60 eV.

**Table S1** Determination result of 8 lignans in SC (n=3)

| **No.** | **Compound** | **Batches** | | | **Mean%** | **RSD%** |
| --- | --- | --- | --- | --- | --- | --- |
|  |  | **1** | **2** | **3** |  |  |
| 1 | Schizandrin | 0.5778 | 0.5846 | 0.5751 | 0.5792 | 0.85 |
| 2 | Gomisin J | 0.0540 | 0.0547 | 0.0537 | 0.0541 | 1.02 |
| 3 | Schisandrin B | 0.1995 | 0.2022 | 0.1977 | 0.1998 | 1.15 |
| 4 | Gomisin G | 0.0257 | 0.0262 | 0.0266 | 0.0262 | 1.70 |
| 5 | Schisantherrin A | 0.0129 | 0.0130 | 0.0133 | 0.0131 | 1.60 |
| 6 | Deoxyschisandrin | 0.1299 | 0.1302 | 0.1291 | 0.1297 | 0.44 |
| 7 | Schisandrin B | 0.3836 | 0.3839 | 0.3801 | 0.3825 | 0.55 |
| 8 | Schisandrin C | 0.0643 | 0.0647 | 0.0638 | 0.0642 | 0.71 |

**Table S2** Summary of the differential metabolites between Control and Model group in plasma samples (Positive ion mode)

| No. | *m/z* _ Rt | T^a^ | T^b^ | No. | *m/z* _ Rt | T^a^ | T^b^ |
| --- | --- | --- | --- | --- | --- | --- | --- |
| 1* | 758.5713_15.29 | ↑ | ↓ | 30 | 564.3600_25.52 | ↑ | ↑ |
| 2* | 782.5718_15.29 | ↑ | ↓ | 31 | 542.3253_8.16 | ↑ | ↓ |
| 3 | 524.3721_12.26 | ↑ | ↓ | 32 | 781.5551_18.55 | ↑ | ↓ |
| 4* | 806.5693_15.30 | ↑ | ↓ | 33 | 570.3566_9.79 | ↑ | ↓ |
| 5* | 758.5700_25.12 | ↑ | ↓ | 34 | 811.6045_17.81 | ↑ | ↓ |
| 6 | 568.3403_9.23 | ↑ | ↓ | 35 | 754.5388_14.36 | ↑ | ↓ |
| 7* | 780.5563_25.22 | ↑ | ↑ | 36 | 546.3472_9.25 | ↑ | ↓ |
| 8* | 759.5763_25.15 | ↑ | ↓ | 37 | 766.5750_15.50 | ↑ | ↓ |
| 9* | 432.2809_7.01 | ↓ | ↓ | 38* | 362.3274_5.83 | ↑ | ↓ |
| 10 | 758.5702_17.80 | ↑ | ↓ | 39 | 494.3253_7.93 | ↓ | ↑ |
| 11* | 806.5694_25.24 | ↑ | ↓ | 40 | 786.6023_18.01 | ↑ | ↓ |
| 12 | 1047.7406_12.16 | ↑ | ↓ | 41 | 373.2748_4.26 | ↑ | ↑ |
| 13 | 546.3538_12.71 | ↓ | ↑ | 42 | 703.5771_17.15 | ↑ | ↓ |
| 14 | 834.6000_25.14 | ↑ | ↓ | 43 | 546.3533_11.86 | ↑ | ↑ |
| 15* | 828.5517_25.21 | ↑ | ↓ | 44 | 520.3394_8.70 | ↑ | ↑ |
| 16 | 1087.6732_9.25 | ↑ | ↓ | 45 | 524.3722_12.77 | ↓ | ↓ |
| 17 | 810.5985_25.12 | ↑ | ↑ | 46 | 510.3562_10.63 | ↑ | ↑ |
| 18 | 832.5853_25.22 | ↑ | ↑ | 47 | 732.5564_25.32 | ↑ | ↓ |
| 19 | 1063.6735_9.22 | ↑ | ↓ | 48 | 822.6015_14.86 | ↑ | ↓ |
| 20 | 761.5900_17.79 | ↓ | ↑ | 49 | 415.2122_6.89 | ↓ | ↓ |
| 21 | 496.3398_9.47 | ↑ | ↑ | 50 | 400.3440_11.76 | ↑ | ↓ |
| 22 | 274.2744_5.74 | ↑ | ↓ | 51 | 433.2811_25.53 | ↑ | ↓ |
| 23 | 546.3566_10.14 | ↑ | ↓ | 52 | 754.5387_25.28 | ↑ | ↓ |
| 24 | 808.5840_25.20 | ↓ | ↑ | 53 | 548.3727_11.24 | ↑ | ↓ |
| 25* | 804.5538_25.22 | ↑ | ↓ | 54 | 433.2863_15.99 | ↓ | ↑ |
| 26 | 991.6725_9.47 | ↑ | ↑ | 55 | 453.1684_6.99 | ↓ | ↓ |
| 27 | 357.2793_7.48 | ↑ | ↑ | 56 | 876.5712_25.23 | ↓ | ↑ |
| 28* | 318.3011_5.66 | ↑ | ↓ | 57 | 802.5368_25.18 | ↑ | ↓ |
| 29 | 796.5872_17.02 | ↓ | ↓ | 58 | 808.5841_18.36 | ↑ | ↓ |

**Table S3** Summary of the differential metabolites between Control and Model groups in plasma samples (Negative ion mode)

| No. | *m/z* _ Rt | T^a^ | T^b^ | No. | *m/z* _ Rt | T^a^ | T^b^ |
| --- | --- | --- | --- | --- | --- | --- | --- |
| 1* | 802.5558_13.83 | ↑ | ↑ | 16 | 329.2483_14.70 | ↑ | ↓ |
| 2* | 766.5373_12.21 | ↑ | ↑ | 17 | 762.5050_16.06 | ↑ | ↑ |
| 3 | 453.2837_4.62 | ↑ | ↑ | 18 | 310.8781_27.72 | ↑ | ↓ |
| 4 | 453.2845_5.81 | ↑ | ↑ | 19* | 803.5602_9.79 | ↑ | ↓ |
| 5* | 852.5704_12.55 | ↑ | ↓ | 20 | 592.2991_11.30 | ↑ | ↑ |
| 6 | 78.9629_26.59 | ↑ | ↓ | 21* | 579.3897_16.99 | ↓ | ↑ |
| 7 | 437.2892_7.76 | ↑ | ↑ | 22* | 552.3085_9.57 | ↑ | ↓ |
| 8 | 802.5566_16.27 | ↑ | ↓ | 23* | 850.5629_25.61 | ↑ | ↓ |
| 9 | 407.2781_4.91 | ↑ | ↑ | 24* | 830.5872_25.59 | ↓ | ↑ |
| 10 | 391.2839_8.02 | ↑ | ↑ | 25 | 863.5564_15.42 | ↑ | ↓ |
| 11 | 640.2982_9.41 | ↑ | ↓ | 26 | 854.5869_25.61 | ↑ | ↑ |
| 12 | 802.5577_25.58 | ↑ | ↓ | 27 | 843.5522_14.51 | ↑ | ↓ |
| 13 | 96.9668_26.54 | ↑ | ↓ | 28 | 265.1475_7.98 | ↑ | ↓ |
| 14 | 485.2797_12.84 | ↓ | ↑ | 29 | 162.8388_27.26 | ↓ | ↑ |
| 15 | 889.5720_15.54 | ↑ | ↓ | 30* | 826.5626_25.63 | ↑ | ↓ |

**Table S4** Summary of the differential metabolites between control and model groups in bile samples (Positive ion mode)

| No. | *t*_R_/min | *m/z* | T^a^ | T^b^ | No. | *t*_R_/min | *m/z* | T^a^ | T^b^ |
| --- | --- | --- | --- | --- | --- | --- | --- | --- | --- |
| 1 | 2.97 | 445.1958 | ↑ | ↓ | 19 | 2.27 | 518.0305 | ↑ | ↑ |
| 2 | 24.48 | 496.4250 | ↑ | ↓ | 20 | 14.94 | 483.3013 | ↑ | ↓ |
| 3 | 7.90 | 434.2815 | ↓ | ↓ | 21 | 6.77 | 301.1230 | ↑ | ↑ |
| 4 | 23.61 | 179.0718 | ↑ | ↓ | 22 | 2.25 | 520.0277 | ↑ | ↓ |
| 5 | 23.43 | 394.3558 | ↑ | ↓ | 23* | 6.47 | 445.1953 | ↑ | ↓ |
| 6***** | 2.20 | 141.0198 | ↑ | ↓ | 24* | 2.38 | 345.9906 | ↑ | ↓ |
| 7 | 4.34 | 305.1543 | ↑ | ↑ | 25* | 2.34 | 397.1530 | ↓ | ↓ |
| 8 | 4.68 | 234.1524 | ↑ | ↓ | 26 | 2.38 | 342.9996 | ↑ | ↑ |
| 9 | 3.16 | 233.0954 | ↑ | ↓ | 27 | 12.34 | 315.1391 | ↑ | ↓ |
| 10 | 2.20 | 159.0308 | ↑ | ↑ | 28 | 3.23 | 317.1298 | ↑ | ↑ |
| 11 | 24.64 | 338.3455 | ↑ | ↑ | 29 | 21.95 | 205.0882 | ↑ | ↓ |
| 12* | 4.78 | 429.2011 | ↑ | ↑ | 30 | 26.86 | 301.1456 | ↑ | ↓ |
| 13 | 2.22 | 353.0762 | ↑ | ↑ | 31 | 6.26 | 301.1233 | ↑ | ↑ |
| 14 | 2.38 | 177.0414 | ↑ | ↑ | 32 | 4.35 | 346.1914 | ↓ | ↓ |
| 15* | 7.73 | 408.2643 | ↓ | ↑ | 33 | 2.38 | 343.9938 | ↑ | ↓ |
| 16 | 4.53 | 170.0623 | ↑ | ↓ | 34* | 4.35 | 369.1576 | ↓ | ↑ |
| 17 | 3.81 | 530.2848 | ↓ | ↑ | 35 | 2.38 | 341.9965 | ↑ | ↑ |
| 18 | 2.38 | 344.9968 | ↑ | ↓ | 36* | 13.48 | 561.3630 | ↑ | ↑ |

**Table S5** Summary of the differential metabolites between control and model groups in bile samples (Negative ion)

| No | *m/z* _ Rt | T^a^ | T^b^ | No | *m/z* _ Rt | T^a^ | T^b^ |
| --- | --- | --- | --- | --- | --- | --- | --- |
| 1 | 1029.5676_12.53 | ↓ | ↑ | 15 | 326.9464_2.45 | ↑ | ↓ |
| 2 | 648.9064_2.46 | ↑ | ↓ | 16 | 511.1685_3.63 | ↑ | ↓ |
| 3 | 650.9037_2.46 | ↑ | ↓ | 17 | 498.2869_11.99 | ↓ | ↓ |
| 4* | 514.2867_12.60 | ↓ | ↑ | 18 | 654.8978_2.46 | ↑ | ↓ |
| 5* | 496.2739_12.26 | ↑ | ↓ | 19* | 517.2867_13.25 | ↓ | ↓ |
| 6* | 646.9096_2.46 | ↑ | ↓ | 20* | 498.2891_15.09 | ↑ | ↑ |
| 7 | 652.9014_2.46 | ↑ | ↓ | 21 | 1028.5469_9.95 | ↓ | ↑ |
| 8* | 512.2697_9.69 | ↓ | ↑ | 22 | 404.9061_3.19 | ↑ | ↓ |
| 9 | 508.2397_14.01 | ↑ | ↓ | 23* | 498.2888_12.66 | ↓ | ↑ |
| 10* | 1025.5408_9.99 | ↑ | ↑ | 24 | 500.2930_15.47 | ↓ | ↑ |
| 11* | 530.2670_14.75 | ↑ | ↓ | 25 | 516.2824_7.26 | ↑ | ↓ |
| 12* | 514.2717_17.79 | ↑ | ↓ | 26 | 496.2766_14.97 | ↓ | ↓ |
| 13* | 510.2522_11.40 | ↑ | ↑ | 27 | 324.9495_2.45 | ↑ | ↓ |
| 14* | 254.0756_5.86 | ↑ | ↑ | 28 | 1011.5568_12.30 | ↑ | ↓ |

T^a^: Model *vs* Control group; T^b^: SC *vs* Model group.

*There were significant differences between the SC and Model group (P < 0.05).


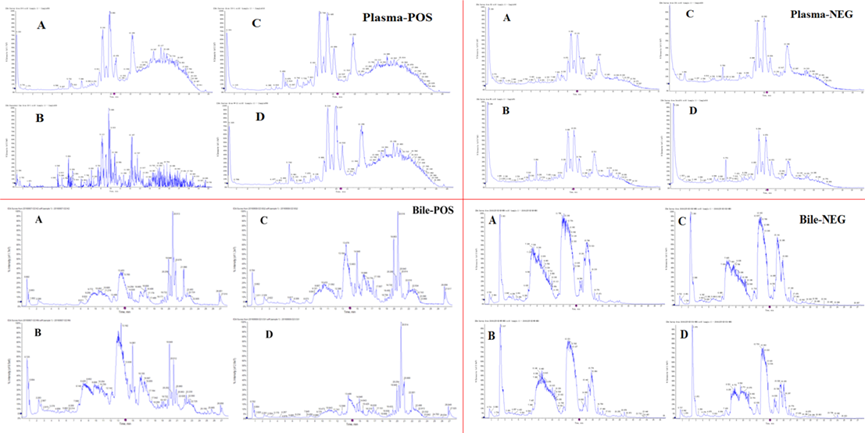


**Figure S1** Typical total ion chromatogram (TIC) of the rat in each group plasma and bile obtained in ESI positive and negative mode based on UPLC-Q-TOF/MS. A: Control group; B: Model group; C: SC group; D: Silymarin group.
